# Projected climate suitability for Hungarian tourism in the 21st century: application of the Holiday Climate Index and modified Tourism Climate Index

**DOI:** 10.1007/s00484-025-02901-y

**Published:** 2025-04-16

**Authors:** Attila Kovács, Gergely Molnár, Otília A. Megyeri-Korotaj

**Affiliations:** 1 Department of Atmospheric and Geospatial Data Sciences, University of Szeged, 2 Egyetem Str, Szeged, HU-6722 Hungary; 2 National Laboratory for Water Science and Water Safety, Unit of Climate Research, HungaroMet Hungarian Meteorological Service, 1 Kitaibel Pál Str, Budapest, HU-1024 Hungary

**Keywords:** Climate change, Tourism, Climate modelling, Holiday Climate Index (HCI), Modified Tourism Climate Index (mTCI), Hungary

## Abstract

Climatic factors significantly influence travellers’ destination selection and demand for tourism products. To maintain sustainable tourism development, it is essential to monitor how tourist destinations will be affected by the projected future changes in the climate system. This study aims to reveal the future impact of climate change for tourism sector in Hungary using the Holiday Climate Index specified for urban tourism (HCI-Urban) and a modified version of Tourism Climate Index (mTCI). The paper employs an ensemble of multiple climate models (ALADIN5.2 and REMO2015) and emission scenarios (RCP4.5 and RCP8.5) to determine the future evolution of indices. Initially, the reference climatic conditions derived from the observational dataset CarpatClim-HU are presented. Then, the climate model results are validated against the observational database for both indices. Afterwards, bias-corrected model outputs are analysed for the periods 2041–2070 and 2071–2100 based on the ensemble of models and scenarios. The spatial distributions of indices are depicted on a monthly level and at a Hungarian district spatial scale. Both the observational and model results highlight that the climatic conditions are more favourable in spring and autumn compared to the summer months. It is also demonstrated that the climatic conditions in Hungary are expected to be more favourable or unchanged from autumn until spring, while there is a general worsening in summer. In terms of the future directions, larger uncertainties are found in May and September. Based on the anticipated trends, the paper pinpoints sector-specific recommendations for adapting tourism services to the altered climatic conditions.

**Supplementary Information:**

The online version contains supplementary material available at 10.1007/s00484-025-02901-y.

## Introduction

Tourism is generally an important driver of the economy. The contribution of tourism-specific activities to the Hungarian economy reached 6% of gross domestic product in 2022, while it was 8.8% considering multiplier production effects. Additionally, tourism industries directly generated 8.9% of national economy employment in 2022 (HCSO 2023). The primary motivation of multi-day domestic trips in Hungary is entertaining and relaxation, followed by visiting friends or relatives, and health preservation (HCSO 2024a). The overnight inbound trips to Hungary are particularly motivated by leisure and entertainment (e.g., sightseeing and vacation), as well as by visiting relatives and friends (HCSO 2024b). Since these activities can also be conducted outdoors, it can be inferred that they are significantly exposed to the prevailing weather and climatic conditions. Additional basic aspects of Hungarian tourism are summarised in Table S1.

Weather and climate have long been recognised as influencing tourism volumes, either promoting or constraining tourism activities (Smith 1993; Perry 1997; de Freitas 2003). Certain activities, like coastal and winter tourism, water sports, or health tourism for climate therapy, depend heavily on weather or climatic factors. For others, such as sightseeing and hiking, weather and climate serve as complementary resources, influencing the success of these activities (Smith 1993; Fagence and Kevan 1998; Gómez Martín 2005). Climate, alongside other resources, plays a significant role in travellers’ motivations and destination choices (Hamilton and Lau 2005; Gössling et al. 2006; Moreno 2010; Scott and Lemieux 2010). Hence, to maintain a sustainable tourism development, it is indispensable to monitor how tourist destinations will be impacted by the anticipated transformation of the climate system in the future.

Climate change is a major challenge for the highly sensitive and vulnerable tourism industry (Gössling et al. 2012; Scott et al. 2012; TPCC 2023). Projected shifts in climate resources are expected to directly amend both domestic and international tourism flows, affecting tourism demand and supply (Rutty and Scott 2014; Scott et al. 2016). As a result, certain destinations may gain competitive advantage, while others lose their prominence in the market (Scott et al. 2004; Aygün Oğur 2023). Additionally, tourism is indirectly affected by the broader environmental impacts of climate change (e.g., sea level rise, snow availability, biodiversity loss), which poses a substantial barrier to tourism growth (UNWTO 2008; Scott et al. 2012; TPCC 2023).

The latest report of the Intergovernmental Panel on Climate Change outlines a shift in future tourism potential across Europe. Precisely, Northern Europe is expected to see more pleasant climatic conditions from May to October, while Southern Europe may experience declines in summer (Bednar-Friedl et al. 2022). Due to its geographical location between Northern and Southern Europe, Hungary can be characterised by a transition in the seasonal tourism climate trends (Kovács 2017). A recent study evaluating twenty-six climate simulations for Hungary indicated consistent temperature growth for all seasons, ranging from 1.3 °C to 3.3 °C under RCP4.5 emission scenario (Representative Concentration Pathways; Moss et al. 2010) and 3.2 °C to 5.7 °C under RCP8.5 by the end of this century (Bán et al. 2021b). Another study, based on four simulations used as inputs in current paper, predicted a temperature increase between 1.6 °C and 2.9 °C under RCP4.5, and 3.4 °C to 4.0 °C under RCP8.5 (Megyeri-Korotaj et al. 2023). Regarding precipitation, both studies found increases in winter, spring, and autumn, while trends remained uncertain in summer. Uncertainty in summer precipitation in Hungary also arises from its location in a transitional zone between Northern Europe with precipitation increase and Southern Europe with precipitation decrease.

The impact of climate on tourism can be quantitatively analysed using different special climate indices (Matzarakis 2007; de Freitas et al. 2008). From the over two hundred climate indices in climatology and biometeorology (Demiroglu et al. 2020; Rutty et al. 2020), many have been used for tourism (e.g., Effective Temperature, or Physiologically Equivalent Temperature), reflecting the difficulty of creating comprehensive indices. Most of these metrics, however, focus solely on thermal aspects (i.e., air temperature and humidity, and some also considering wind speed and radiation). As tourists experience the integrated effects of the atmospheric environment, composite indices are needed to also account for aesthetic (sunshine/cloudiness) and physical (precipitation and wind) elements (de Freitas 2003; de Freitas et al. 2008). These indices range from simple numeric values to more complex evaluation schemes, such as the Climate Index for Tourism (de Freitas et al. 2008; Bafaluy et al. 2014) and the Climate-Tourism-Information-Scheme (CTIS; Matzarakis 2007).

The concept of a simple index was established as Mieczkowski’s Tourism Climate Index (TCI; Mieczkowski 1985). In TCI, the tourism-relevant parameters (i.e., air temperature, humidity, sunshine duration, precipitation, and wind speed) are rated, weighted, and summed to produce an overall score, with higher values indicating more favourable conditions for outdoor tourism activities. Over time, TCI has become the most popular index due to its simplicity, reliance on easily accessible monthly meteorological data, and ability to compare the climate potential of different destinations (e.g., Scott et al. 2004; Perch-Nielsen et al. 2010; Adiguzel et al. 2022). However, TCI has faced criticism for its expert-based construction, particularly the subjective rating and weighting schemes that do not reflect tourists’ stated climatic preferences or tourism performance data (e.g., visitation). Other limitations include its additive structure, which fails to account for the overriding effect of intensive precipitation and wind, and its monthly temporal resolution, which is too coarse given tourists’ daily sensitivity to weather. Furthermore, TCI was developed for general outdoor tourism and does not address the specific requirements of different tourism types, such as urban, beach, or winter sports tourism (Scott et al. 2016; Rutty et al. 2020).

The deficiencies of TCI inspired researchers to develop improved composite indices. The Holiday Climate Index (HCI) overcomes these limitations by relying on tourists’ stated preferences from surveys, using daily data, and considering the overriding effect of precipitation and wind. Additionally, HCI is tailored for two tourism segments: urban (HCI-Urban; Scott et al. 2016) and beach tourism (HCI-Beach; Rutty et al. 2020). Currently, besides TCI, it has become the most widely used index, describing climate suitability for urban environments (Hasanah et al. 2020; Velea et al. 2022, 2023; Aygün Oğur 2023), beach destinations (Matthews et al. 2021; Yu et al. 2021), or both (Demiroglu et al. 2020; Carrillo et al. 2022; Ma et al. 2023; Samarasinghe et al. 2023; Bilgin et al. 2024; Prinsloo and Fitchett 2024). Building on the seminal TCI, other promising indices, such as the CIT, Beach Climate Index (BCI; Morgan et al. 2000), modified TCI (mTCI; Kovács et al. 2016, 2017), Camping Climate Index (CCI; Ma et al. 2020), or optimised Holiday Climate Index (Matthews et al. 2021), have been developed to overcome TCI’s limitations. Some tourism indices, including HCI, have been incorporated into climate indicators provided by Copernicus Climate Change Service for the European tourism sector (C3S 2024).

Studies investigating climate potential specialised for tourism are subject to limited knowledge in Hungary. From the quantification of the recent tourism climate conditions (Kovács and Unger 2014a, b; Kovács et al. 2016), researches have increasingly turned to explore the expected future changes (Kovács 2017; Kovács et al. 2017; Sütő and Fejes 2019; Kovács and Király 2021). However, the most recent climate projections specialised for Hungary were not yet included in tourism context analyses. Using conventional and increasingly advanced tourism climate metrics, investigations have focused on certain popular Hungarian and European tourism locations (Kovács and Unger 2014a, b; Kovács et al. 2016; Kovács 2017), as well as have pertained to entire Hungary (Kovács 2017; Kovács et al. 2017; Sütő and Fejes 2019; Kovács and Király 2021). By doing so, two most popular tourism climate evaluation tools (TCI and CTIS) were preferred during the calculations, which have been updated using thermal perception and preference surveys from Hungary (mTCI and modified CTIS; Kovács et al. 2016, 2017; Kovács 2017). The most recent index (i.e., HCI) was not yet incorporated in the corresponding studies.

To bridge some of the aforementioned gaps, this paper aims to implement the HCI-Urban and mTCI indices to reveal the future impact of climate change for tourism in Hungary. The inclusion of the Holiday Climate Index is major novelty of this study, since this is the first use of this specific index in Hungary. The other applied index (mTCI) was developed by the first author (Kovács et al. 2016, 2017) and is also included to overcome most limitations of TCI. It is utilised in comparison to HCI to present the uncertainties among indices, and to show the differences relative to previous studies in which the index was initially introduced.

Furthermore, this paper focuses on incorporating the latest regional climate model (RCM) results specialised for Hungary. The study employs a multi-model, multi-scenario, and multi-index technique to interpret the uncertainties arising from different RCM projections and their drivers (global climate models), from the choice of scenarios, and from the selection of indices. With this set of models, the basis for other impact assessments and thus better coherence between the different impact assessments and adaptation plans are intended to be facilitated for Hungary.

In order to fulfil the research goals outlined above, the scenario-specific RCM projections for the tourism climate indices are corrected with the delta method, based on a gridded database of observations developed in Hungary. The corrected results are presented for two thirty-year climate periods: 2041–2070 and 2071–2100. In contrast with our previous work (Kovács and Király 2021), monthly averages are replaced by daily data to refine the calculation method of indices. Nevertheless, for simplicity, the spatial distributions of indices are displayed in an aggregated form, on a monthly level and at Hungarian district scale. Instead of presenting outcomes for each model experiment individually, the results for the ensemble of models and scenarios are displayed in the form of quantile maps. With the related maps, the minimum, median, and maximum of gridded index data for each month and future period are specified to properly interpret the uncertainties concerning distinct climate projections. Following the presentation of the results, an insightful discussion is provided that highlights the findings of this paper with respect to international research. This section also offers sector-specific recommendations for adapting tourism services to the altered climatic conditions, based on the anticipated future trends.

## Materials and methods

### Tourism climate indices

The indices applied in this study (mTCI and HCI-Urban, the latter hereinafter referred to as HCI) are derived from the theory of TCI but improve its main deficiencies. TCI evaluates the climate’s impact on general outdoor activities (e.g., sightseeing, recreation, and other light physical activities), while mTCI and HCI focus on urban tourism. The TCI’s basic meteorological parameters, sub-indices, and their rating scores and weights are summarised in Table S2 (Mieczkowski 1985). TCI uses monthly metrics of daily variables (i.e., monthly means, and sums for precipitation) with assigned rating scores (highest: optimal, lowest: unfavourable). The ratings of *CId* and *CIa* sub-indices characterise outdoor thermal comfort, based on Effective Temperature (ET), an early and simple empirical index using air temperature and humidity (Houghten and Yaglou 1923). The composite TCI value is the sum of the sub-index scores, weighted by their relative importance, as given in Eq. 1:

1 $$\:\text{TCI}/\text{mTCI}=2(4CId+CIa+2P+2S+W)$$

Unlike TCI, the mTCI index employs daily climatic parameters instead of monthly ones. Moreover, the thermal comfort sub-indices (*CId* and *CIa*) have been improved by replacing ET with the Physiologically Equivalent Temperature (PET; Höppe 1999) index (Kovács et al. 2016). PET has been the most widely used thermal comfort index, accounting for all relevant climatic parameters (air temperature and humidity plus wind speed and thermal radiation) and personal factors (e.g., clothing and activity). Since PET’s rating schemes were empirically tested against human perception, mTCI offers a more accurate estimate of thermal comfort conditions.

The developed PET rating schemes are based on the seasonally different thermal perception patterns of Hungarian residents (Kovács et al. 2016, 2017). PET scores were determined from an extensive (with 5805 items) outdoor thermal comfort (OTC) survey conducted in Szeged, Hungary (Kántor et al. 2016; Kovács et al. 2016). The survey spanned from spring to autumn; winter was excluded from this analysis. Consequently, the PET rating scores range between 0 and 5, varying by season (spring, summer, autumn). The rating system of the *P*, *S*, and *W* components, the sub-index weights, and the calculation formula (Eq. 1) remained unchanged in the modified index. Basic information on mTCI construction are in Table S3.

The other applied index, HCI, is also used to overcome TCI’s main deficiency, which involves arbitrary rating scores and weights of sub-indices (Scott et al. 2016). Furthermore, HCI considers the overriding effect of precipitation and wind by adding amended rating scores (even negative values) and weights for the *P* and *W* sub-indices. Finally, like mTCI, HCI relies on daily meteorological data. In HCI, the thermal comfort sub-index was originally defined by effective temperature (Scott et al. 2016), but recent studies (Demiroglu et al. 2020; Rutty et al. 2020; Matthews et al. 2021; Aygün Oğur 2023; Samarasinghe et al. 2023; Prinsloo and Fitchett 2024) preferred the Canadian Humidex formula (Eq. 2; Burke et al. 2006). Humidex requires air temperature (*T*, °C) and relative humidity (*RH*, %) for calculation (Eq. 2). Table S4 summarises the parameters, rating scores, and weights applied in HCI. Like previous indices, the weighted scores are combined into a single value. Thus, HCI is calculated using Eq. 3.

2 $$\:\text{Humidex}=T+\frac{5}{9}\times\:\left(\left(6.112\times\:1{0}^{\left(\frac{7.5\times\:T}{237.7+T}\right)}\times\:\frac{RH}{100}\right)-10\right)$$

3 $$\:\text{HCI}=4TC+2A+3P+W$$

The overall index score of TCI, mTCI, and HCI commonly ranges between 0 and 100. Based on predefined score scales, descriptive categories were established. This qualitative categorisation is more user-friendly compared to raw index values. In every case, higher score ranges indicate more favourable conditions for tourism activities. For mTCI, the evaluation system remained unchanged from TCI (Table S5).

Complete description on TCI construction, including the individual sub-index rating systems, are presented in Mieczkowski (1985) and Kovács et al. (2016, 2017). Detailed conceptual and methodological aspects of mTCI development are available in Kovács et al. (2016, 2017), while the applied form of HCI is introduced in Scott et al. (2016).

### Databases and calculations

This study aims to determine the future evolution of mTCI and HCI indices for Hungary. The reference climatic conditions are characterised for the climate period 1971–2000, using observational database CarpatClim-HU developed by the HungaroMet Hungarian Meteorological Service (Bihari et al. 2017). The database comprises grid point data for Hungary, with a horizontal spatial resolution of 0.1° × 0.1° (approx. 10 km), covering latitudes from 45.8°N to 48.5°N and longitudes from 16.2°E to 22.8°E. This represents 1,104 grid points across the country (Fig. S1). The grid values were derived from controlled homogenised meteorological measurement data, interpolated to the 0.1° resolution grid and harmonised along national borders.

Climate model data were obtained from the REMO2015 and ALADIN 5.2 regional climate models, driven by different global climate models (Table S6). These RCMs are the latest model results implemented by HungaroMet. Further details on these models are found in Bán et al. 2021a, b; Suga et al. (2021); and Megyeri-Korotaj et al. (2023). Data were available on the same grid as the observational database (Fig. S1). For both RCMs, two emission scenarios, RCP4.5 and RCP8.5, described future anthropogenic activity (Table S6). These scenarios differ in greenhouse gas concentration trajectories. RCP4.5 is an intermediate scenario, stabilising radiative forcing at 4.5 W/m^2^ by 2100 compared to pre-industrial conditions. In RCP8.5, emissions rise throughout the 21st century, to a radiative forcing of 8.5 W/m^2^ by century’s end. RCP8.5 is considered a ‘worst-case’ scenario with no mitigation policy.

For the calculation of mTCI and HCI, daily values of the necessary meteorological parameters were used according to Tables S1 and S4. The daily minimum relative humidity values for mTCI were not directly available in the observational and climate model datasets; thus, they were derived using the method in Whittlesea and Amelung (2010). The daily sunshine duration for mTCI was also missing in the climate models, so it was derived from cloud coverage using the method in Amelung (2006). The PET index, a key component of mTCI, was computed using daily mean cloud cover data and the above-mentioned variables (Table S3) with the RayMan model (Matzarakis et al. 2010). For the *TC* sub-index in HCI, the Humidex formula (Eq. 2) was applied using daily maximum air temperature and mean relative humidity (Table S4).

The results for future conditions are analysed for the climate periods 2041–2070 and 2071–2100. Similar to the observational database, the reference period of the model experiments was 1971–2000. To eliminate systematic model errors in global and regional climate model projections, corrected model results generated with the delta method (Hawkins et al. 2013) were employed. This method involved calculating for each model grid point the changes between future index values (2041–2070, 2071–2100) and values for the model reference period (1971–2000), then adding these changes to the observed data from the same reference period (CarpatClim-HU, 1971–2000). This method was applied to the calculated tourism climate indices (mTCI, HCI), not to each measured and modelled meteorological variable.

The spatial distributions of indices were aggregated on maps for Hungary at a monthly level and district spatial scale. As the smallest administrative-territorial entities in Hungary, districts provide optimal level for analysis, yielding valuable insights for tourists, professionals, and stakeholders. The mTCI results were produced from March to November, while HCI maps were created for the entire year. The results are presented as an ensemble of model experiments, not individually for each model experiment (e.g., REMO RCP4.5). This is interpreted through quantile maps by specifying the minimum, median, and maximum values of the gridded model data.

For this process, first, the monthly means of the daily index data were calculated for each grid point, regarding the observational and corrected future data. Then, the minimum, median, and maximum of the grid point data of all four scenarios (REMO RCP4.5, REMO RCP8.5, ALADIN RCP4.5, ALADIN RCP8.5) were determined for each future period (2041–2070, 2071–2100) and month. Afterwards, the spatial distribution of these quantiles was plotted on maps for each month at the district scale. Technically, district averages were generated with QGIS software from the grid point data within each district. The outcomes were mapped using the descriptive rating systems of the indices (Table S5). Values below 40 were merged into a single ‘unfavourable’ level for mTCI and ‘unacceptable’ for HCI.

## Results

In this section, initially, the tourism climate conditions for the reference period 1971–2000 are analysed using the distribution of HCI and mTCI indices derived from the observational and climate model datasets. Then, the corrected outputs for the periods 2041–2070 and 2071–2100 are presented based on the quantiles (minimum, median, maximum) of model results. All index ratings are depicted on a monthly level. As mentioned in the methodological section, the analysis of mTCI is applied to the months in spring (MAM), summer (JJA), and autumn (SON), while, for the HCI, winter (DJF) is also included.

### Observational results

**Fig. 1:**
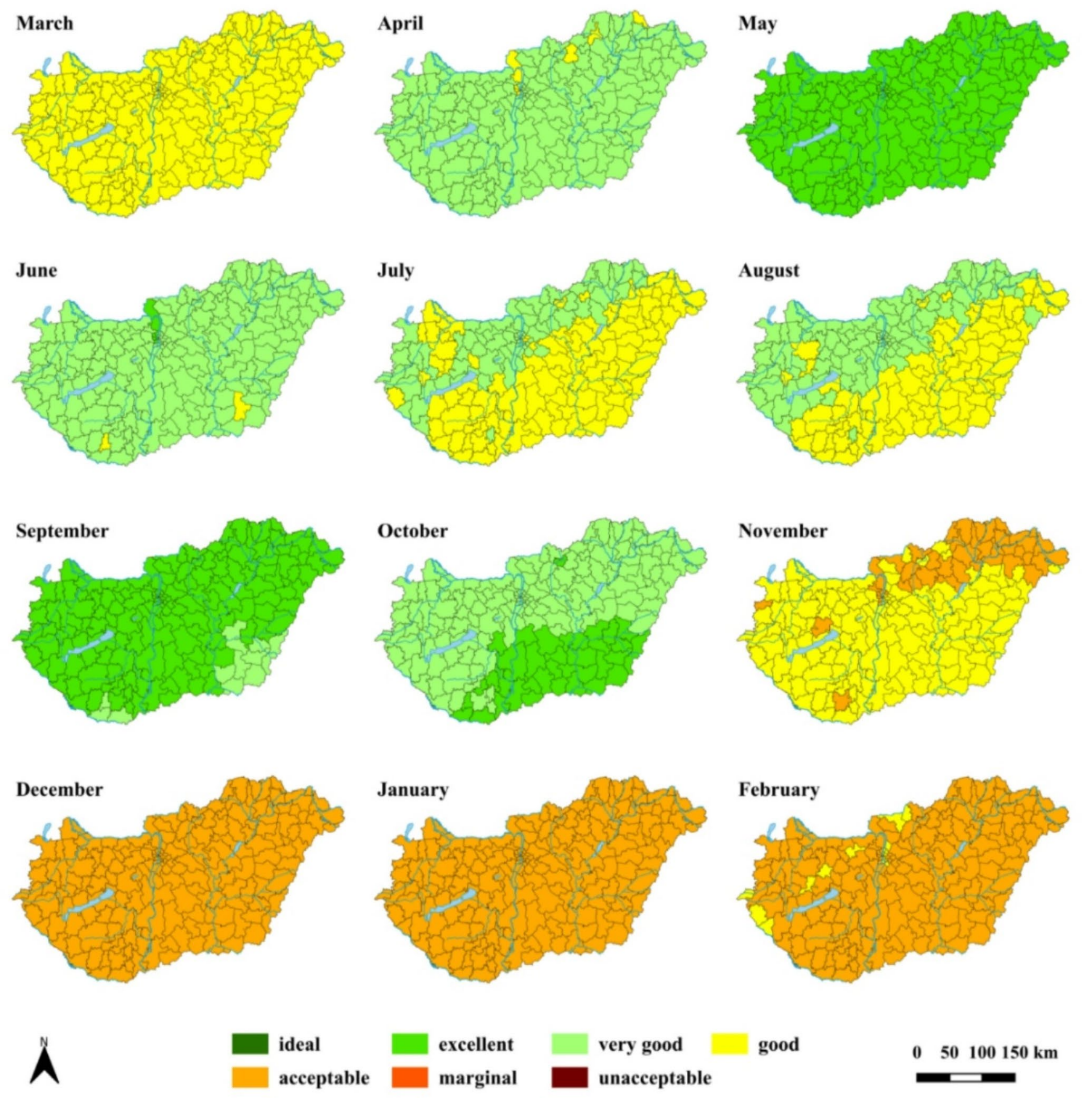
Spatial distribution of monthly HCI ratings by district for the period 1971–2000 based on CarpatClim-HU observational database

According to the classification by Scott and McBoyle (2001), who introduced the potential annual patterns of the TCI, a ‘bimodal’ distribution is outlined for both HCI (Fig. 1) and mTCI (Fig. S2) indices by the observational results for the reference period. This implies that the climatic conditions are more favourable in spring and autumn compared to the summer months when a decline in the climate potential emerges.

Regarding the annual course of HCI, the most unfavourable conditions occur in winter, with ’acceptable’ category in most parts of Hungary. Climatic conditions are becoming more pleasant in spring, peaking in May at ’excellent’ level. In summer, the suitability of climate regresses, particularly in the southeast half of the country characterised with ’good’ conditions. It is important to note that these circumstances still suggest favourable opportunities for urban tourism activities. In September and October, climate potential becomes comparable to May and April, respectively. Afterwards, a strong deterioration is depicted towards the winter period (Fig. 1). The annual course of mTCI is similar to that of HCI, with two peaks appearing in spring and autumn. In general, mTCI signifies slightly less favourable conditions in most months and regions compared to HCI (Fig. S2). The reason for this difference is mainly to be found in the different characteristics in rating systems of the thermal comfort and precipitation sub-indices of HCI and mTCI.

### Climate model validation

In order to identify biases in the regional climate model data, the modelling results were validated by the HungaroMet against the CarpatClim-HU observational database for minimum, mean, and maximum temperatures, precipitation, and various climate indices (Bán et al. 2021a; Megyeri-Korotaj et al. 2023). In the current study, we performed a validation for both tourism climate indices. Generally, it is difficult to measure the effect of individual meteorological variables on the model bias due to the complexity of the indices. However, at both indices, daily maximum temperature is taken into account with one of the highest proportions (40%) and thus it could play a key role in influencing the index ratings.

**Fig. 2:**
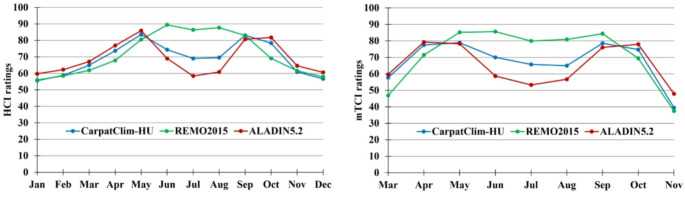
Annual variability of HCI and mTCI ratings for Hungary for the period 1971–2000 based on CarpatClim-HU observational database as well as REMO2015 and ALADIN5.2 simulations

Figure 2 illustrates the annual variability of the indices for Hungary for the period 1971–2000 based on the observational database and climate model simulations. It can be observed that the REMO model overestimates both indices in summer, suggesting more favourable conditions for tourism activities relative to the observations. This overestimation persists in May and September for mTCI. The higher modelled rating values of REMO are also depicted on the monthly maps (Figs. S3 and S4). This overestimation might be caused by the underestimation of daily maximum temperature by REMO model (Megyeri-Korotaj et al. 2023). In contrast to REMO, both indices are underestimated for the summer months by the ALADIN model (Figs. 2, S5 and S6).

Apart from summer, there is a greater agreement between the observed and simulated values, with some underestimations by the REMO and overestimations by the ALADIN model. In case of the HCI given by REMO model, the lowest biases appear in winter as well as in March, May, September and November (Figs. 2, S3). In the meantime, the ALADIN-based outputs result in the least errors during the spring months (Figs. 2, S5). Considering mTCI, the lowest difference occurs in November according to REMO (Figs. 2, S4), while there are minor biases in the majority of the vernal and autumnal months in the ALADIN results (Figs. 2, S6).

Finally, it should be noted that most simulations capture appropriately the ‘bimodal’ annual distribution of indices (Figs. 2, S4–S6) that was apparent in the observations (Figs. 1, 2 and S2). The only exception arises at the outcome of REMO for HCI (Figs. 2, S3) for which the most favourable conditions prevail in summer, yielding ‘summer peak’ distribution according to the term defined by Scott and McBoyle (2001). Moreover, the simulations mostly capture the observations given that mTCI indicates slightly less favourable conditions compared to HCI (Figs. 2, S3–S6). This is the most noteworthy for ALADIN where all months except April show this difference (Figs. 2, S5 and S6).

### Climate projections

**Fig. 3:**
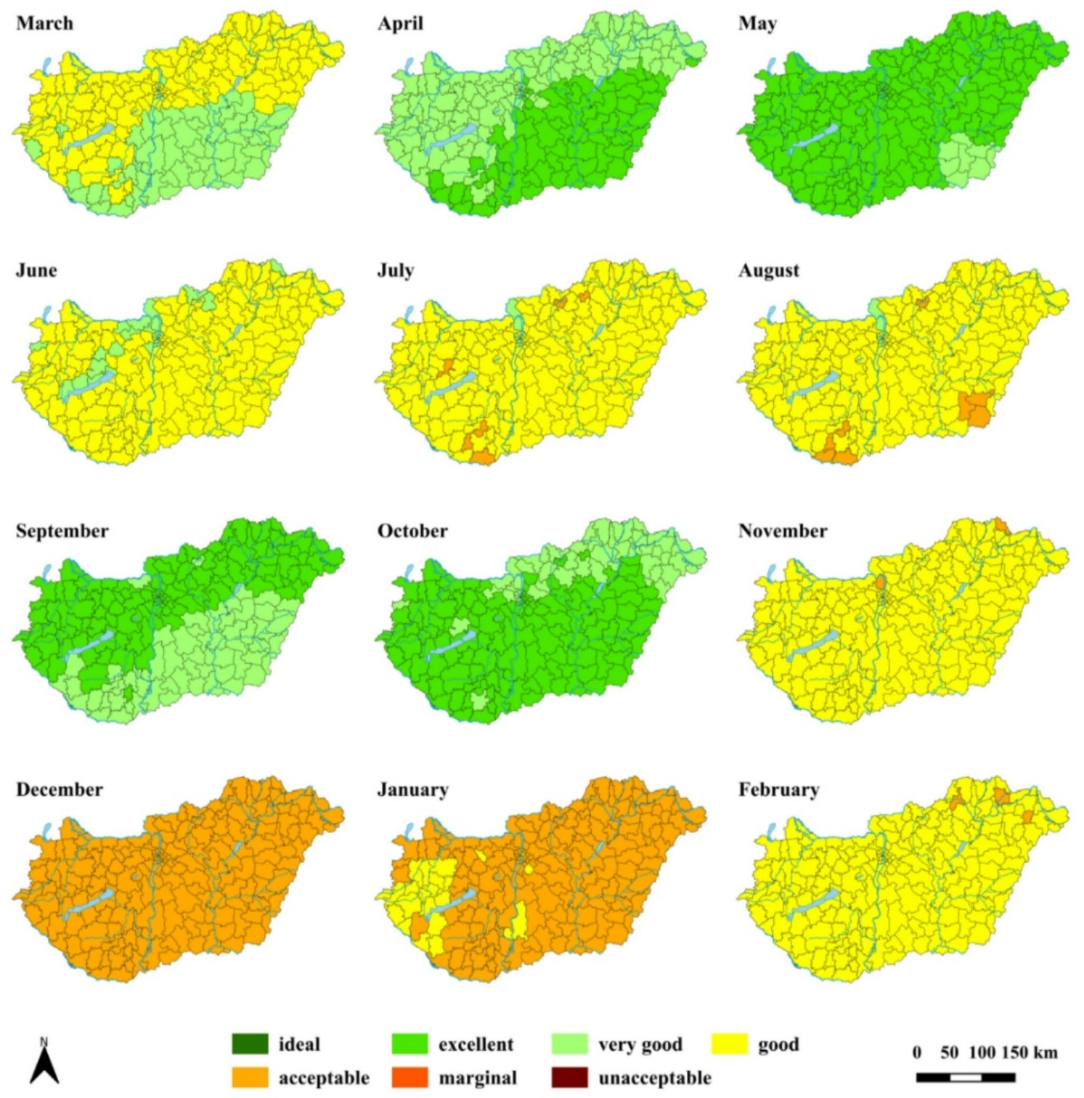
Spatial distribution of monthly HCI ratings by district for the period 2071–2100 based on the median values of simulated results

After identifying the biases between the observed and modelled distributions of HCI and mTCI ratings, the climate projections were bias corrected applying the delta method. For efficiently illustrating the corrected future projections of tourism climate indices, the minimum, median, and maximum values of the four climate model simulations are taken for each grid point into consideration. By doing so, the minimum field can be considered as the ‘worst-case’ scenario for a given index, since the lowest index value among the scenarios represents the least favourable tourism climate conditions (Table S5). On the other hand, the maximum field describes the highest possible value can be reached in the future. The spatial distributions of HCI and mTCI ratings for the period 2071–2100 are displayed in Figs. 3, S7, S8, and S9–S11, respectively, while the results for the period 2041–2070 are available in Figs. S12–S14 and S15–S17 Initially, a general overview is provided for the two future periods, and afterwards, the findings for the later period (2071–2100) are analysed in detail.

#### General characteristics

In accordance with the observational results (Figs. 1, S2), the annual courses of the tourism climate index ratings remain ‘bimodal’ in all future cases; i.e., decline in climate potential can be observed in summer (Figs. 3, S7–S17). It is well documented that the mean temperature is increasing globally as well as in Europe (Kjellström et al. 2018), with a general southward increase in the Carpathian Basin. This pattern appears in the index results as well. For HCI, a northwest-to-southeast pattern is visible (Figs. 3, S7, S8, and S12–S14), while there is a northeast-to-southwest gradient regarding mTCI (Figs. S9–S11, S15–S17). As an example, less favourable conditions are expected from May to September in the southeast parts of the country compared to the other areas according to HCI, whereas this applies to south-western Hungary on the basis of mTCI.

Future tendencies relative to the observations indicate that the climatic conditions for urban tourism are getting more favourable or remaining unchanged from autumn until spring, while in summer, due to the increasing temperature and more frequent heat extremes, the climatic conditions are generally worsening (Figs. 3, S7–S17). The changes are more significant towards the end of the century (Figs. 3, S11–S11). By comparing the indices, it can be concluded that mTCI usually demonstrates slightly worse conditions for tourism in the future than HCI, which indicates a firm consistency with the observational results.

#### Future trends (2071–2100)

In order to highlight the most significant future shifts in the climate potential, subsequently, the results for the period 2071–2100 are analysed in detail (Figs. 3, S7–S11). The corresponding assessment focuses primarily on the differences between the individual months and indices with respect to the future trends.

In winter, the climatic conditions are generally expected to remain ‘acceptable’, however, an improvement to ‘good’ can be realised in February according to each quantile field (Figs. 3, S7–S8), while it is concluded in December and January according to the maximum field (Fig. S8).

In March and April, climatic conditions are getting better by one level from the southeast, based on the median and maximum fields of HCI (Figs. 3, S8). The mTCI signifies improvements in all cases (Figs. S9–S11), which can attain two categories in March, based on the median and maximum fields (Figs. S10–S11). October and November show comparable tendency to April and March, respectively. During these autumnal months, both metrics indicate one-category improvement towards the maximum field (Figs. 3, S7–S11), which can still be greater in November according to mTCI (Figs. S9–S11).

Possible deterioration in climate potential begins to develop in May and September. In May, according to both indices, the climatic conditions will most likely be unaffected, although one-category decline appears in some parts of Hungary (Figs. 3, S7, S9–S11). In September, the quantile fields show larger uncertainties in terms of the possible tendencies, with considerable differences between the indices. The fields of mTCI depict a decline by one level in large parts of the country and no improvement can be noticed in any part of Hungary (Figs. S9–S11). In the HCI ratings, changes can cover three categories, with possible decrease and increase in climatic conditions. According to the minimum field, the suitability of climate regresses everywhere by one or two levels (Fig. S7), while the maximum map demonstrates an improvement to be ‘ideal’ in the northern regions of the country (Fig. S8). According to the median case, the southern parts can undergo a one-level decline, whereas other areas are anticipated to remain at the same conditions (Fig. 3).

In summer, no discernible improvement is expected in any of the analysed indices and fields. Between the ‘best-case‘ (maximum field) to the ‘worst-case‘ (minimum field) scenarios, there is a remarkable decline in the climatic conditions, which spans from northwest to southeast for HCI (Figs. 3, S7–S8) and from northeast to southwest regarding mTCI (Figs. S9–S11). The spatial patterns of the indices illustrate consistency in the degree of the categorical change in the future. Precisely, the maximum maps of HCI and mTCI show one-category decrease or the same conditions (Figs. S8, S11), while the decline reaches two levels in certain parts of the country following the minimum fields (Figs. S7, S9). Furthermore, the median maps mostly depict one-level decrease (Figs. 3, S10). In temporal sense, the most unfavourable conditions are outlined in July and August, although these conditions are still in the ‘acceptable’ or ‘marginal’ levels in the minimum (i.e., pessimistic) case (Figs. S7, S9).

## Discussion and conclusions

This paper aimed to reveal the future impact of climate change for tourism in Hungary using the Holiday Climate Index specified for urban tourism (HCI) and a modified version of the Tourism Climate Index (mTCI). The spatial distributions of indices were depicted on maps, on a monthly level and at a district spatial scale. According to the observations for the period 1971–2000, the annual courses of both indices showed a ‘bimodal’ pattern, which implied that the climatic conditions were more favourable in spring and autumn compared to the summer months. In general, mTCI demonstrated slightly less pleasant conditions in the majority of months and regions compared to HCI. The above features were appropriately captured by the regional climate models ALADIN and REMO for the reference period. By comparing the monthly distribution of the indices from the observations and the simulations, it was observed that the REMO model overestimated while the ALADIN underestimated both indices in summer. In the other seasons, there was a greater agreement between the observed and simulated values.

Bias-corrected future projections were interpreted in the form of minimum, median, and maximum fields for the periods 2041–2070 and 2071–2100. The annual courses of the future index ratings retained the ‘bimodal’ pattern in all cases. The future tendencies relative to the observations indicated more favourable or unchanged conditions from autumn until spring as well as the conditions were found most likely to be deteriorated in summer. The months of May and September showed larger uncertainties in terms of the possible future directions. The changes were more significant towards the end of the century (i.e., period 2071–2100). Considering the indices, similar to the observational results, mTCI usually indicated slightly less favourable future conditions compared to HCI.

In terms of the future tendencies in climate potential, the findings of this paper indicate a firm consistency with several studies. Early analyses using the original TCI index projected consistent changes in climatic conditions for the 21st century in Europe and North America. A decline in climate potential is expected in the Mediterranean region in summer, while better conditions may arise in spring and autumn (and occasionally in winter). In the north parts of Europe and America, however, an improvement can be expected in summer, which may extend to a significant part of spring and autumn (e.g., Scott et al. 2004; Amelung and Viner 2006; Hein et al. 2009; Perch-Nielsen et al. 2010; Amelung and Moreno 2012). The transitional geographical location of Hungary between Northern and Southern Europe explains these trends properly.

Several recent studies using the HCI index presented similar tendencies, being consistent with our results. Demiroglu et al. (2020) evaluated the performance of HCI for the Mediterranean region using RegCM model outputs. In the analysed domain, some parts of Hungary were still included. The results showed some improvement in winter and spring in the observable area, while a deterioration could be explored in summer, particularly in the south part of the country. A research for the Canary Islands using HCI demonstrated that the climate suitability for tourism is likely to improve in autumn, winter and spring, but it may reduce in summer (Carrillo et al. 2022). Velea et al. (2023) concluded that larger areas may experience a higher number of days favourable for tourism in winter and spring in Romania. Aygün Oğur (2023) also found a seasonal shift in HCI for Denizli Province, Turkey. It was concluded that the intermediate seasons will have the most pleasant conditions in the future, moreover, the winter period can also bring some improvements. On the other hand, the climate potential may decrease in summer.

Due to differences in methodologies and result interpretations, making meaningful geographical comparisons of future conditions with the existing literature is challenging. Nevertheless, when comparing our future HCI results with those of Demiroglu et al. (2020), Hungary showed more favourable conditions in this study by 1–3 categories across all seasons. However, in line with our research, they also identified spring and autumn (summer and winter) as the most (least) suitable periods for tourism in Hungary. In Demiroglu et al. (2020), Hungary shares identical winter conditions compared to other European regions, however both Hungary and the northern Balkans face worse conditions in summer than most parts of Europe. This may be attributed to the expected increases in temperature and heat extremes in summer, particularly affecting the Carpathian Basin (Megyeri-Korotaj et al. 2023). In spring and autumn, Hungary’s conditions may align with the European average, though, in these seasons, topographical impacts can cause large regional variations. However, topography does not influence these differences in Hungary (Demiroglu et al. 2020).

It must be highlighted that the expected future trends may provide potential benefits for the Hungarian tourism market, thereby contributing to the development of sustainable tourism in Hungary. The altered climatic conditions make it possible to diversify the tourism supply by extending the tourism season to the spring, autumn, and still the winter seasons (Table 1). In Hungary, the expansion of outdoor lake- and riverside tourism has already been observed for May and September. Furthermore, the climatic conditions can be satisfying for urban tourism in almost the entire spring and autumn, and occasionally in winter. To this trend, the tourism demand has already started to adapt in Hungary. It should be more recognised, however, that the tourism services also have to adapt to the amended demand. Extended opening times of services for the more favourable seasons (e.g., baths, sailing, restaurants, leisure parks) are already observable in the country. For these extended periods, providing services both outdoors (e.g., cultural and gastronomic festivals, themed walks, leisure parks, baths, water parks) and indoors (e.g., festivals, indoor baths and spas, visitor centres, indoor event spaces, medical services) can act as realistic solutions to the changed climatic conditions in Hungary. Indoor activities can also serve as alternative possibilities during cold weather conditions, extreme weather events, as well as in the less favourable summer period. Due to the more frequent heat extremes in summers, the opening time of services and sights (e.g., churches, museums, visitor centres, baths and spas) should be more flexible, for example, by extending the opening hours towards the late evening period (Table 1).

**Table 1:** Recommendations for adapting tourism services to the altered climatic conditions in Hungary

Extension of tourism season to spring, autumn, and winter		
	Suggested services	Suggested actions
Outdoor services for the extended periods	• Cultural and gastronomic festivals • Themed walks • Theme or leisure parks • Baths, spas, water parks	• Extended opening times of outdoor services for several seasons (e.g., baths, sailing, water bikes, restaurants, leisure parks) • More flexible opening hours of indoor services (e.g., churches, museums, visitor centres, baths and spas) • Program proposals for all services • Preparation of outdoor and indoor services for extreme weather events • Consideration of local and microclimatic conditions in choosing the location of outdoor and indoor services
Indoor services for the extended periods and as alternatives	• Cultural and gastronomic festivals • Baths, spas, water parks • Visitor centres • Indoor event spaces • Medical services • Business and conference tourism	

It is crucial to recognise that climate is only one of several factors affecting tourism. The dynamics of tourism are influenced by complex interplay of various natural, social, and economic factors. The tourism climate indices like the ones used in this paper are not able to capture some further weather and climatic factors influencing the suitability of tourism, such as extreme events (e.g., supercells) or urban heat island intensity. Moreover, decisions to travel are determined by several non-climatic factors, particularly institutional ones (e.g., timing of national, religious, and school holidays, or large sporting events), which are important origins of the seasonality in tourism demand. Several social and economic elements (e.g., prior experience, accessibility and distance, budget, safety, epidemics) also influence the tourist flow. For the development of targeted adaptation strategies to climate change, comprehensive impact assessments are required that integrate both climatic and socio-economical drivers into a research framework.

As previously mentioned, several limitations need to be considered when using tourism climate indices to assess the climate suitability for tourism. The temporal and spatial resolutions of these indices are often too coarse to provide meaningful information for decision-making. The additive structure of certain indices, along with the subjective weighting and rating schemes, may not accurately reflect tourists’ actual experiences (de Freitas et al. 2008; Dubois et al. 2016; Scott et al. 2016; Matthews et al. 2021). Regarding subjectivity, Dubois et al. (2016) pointed out that differences in survey samples used to empirically validate the indices (e.g., the survey questions, age groups, and nationalities) can significantly affect the rating scales and thresholds of indices. Additionally, the methods used to compute the indices, including the temporal resolution of raw data and the sequence of averaging and index calculation, play a role in determining the final index value. On the other hand, Ma et al. (2023) highlighted that tourism climate indices may struggle to capture institutional (societal) seasonality, and the correlation between index values and destination visits might be exaggerated due to these institutional factors. As we also demonstrated, in climate change impact assessments, uncertainties in modelling of physical processes as well as in climate model projection and scenario choices substantially affect projections based on climate indices, thereby influencing information for stakeholders (Dubois et al. 2016). Finally, the indices applied in this study focus on the same tourism activity – urban tourism –, which is one of the dominant tourism activities in Hungary. However, other significant types, such as cultural, lake- and riverside tourism, hiking, cycling, and camping, also play a key role. To gain a comprehensive understanding, additional indices that address these activities should be considered, including CCI for camping, CIT for the other activities, and CTIS for a holistic assessment of tourism climate.

Small-scale regional climate models (approx. 10 km), like the ones used in this study, can operate as adequate tools for a country-level examination of the tourism potential by smaller entities (e.g., districts). Information of greater relevance on certain tourist destinations’ potential (e.g., cities and their surroundings, lakeside settlements, mountainous regions), however, can be obtained using surface models with finer resolution (approx. 1 km). These models can provide representative local information for impact assessments in environments with diverse topography or built-up areas. For this reason, a potential development of this research could be the study of climatic conditions of local tourist destinations by comparing outcomes of fine-scale models concerning different scenarios (e.g., emission trajectories and climate indices).

Regardless of the scale of analysis, as previously mentioned, credible comparability of international researches on the climatic impacts on tourism faces substantial challenges. These difficulties arise from the significant differences in the databases used, the diversity of data processing and analysis methods, the various presentation ways of outcomes, and the frequent lack of fundamental methodological information in publications (Kovács and Király 2021). Nevertheless, it can be inferred that the findings of this research extend beyond the country, potentially applying to other European countries with similar climatic conditions and tourism potential. To enhance the relevance and utility of such research, it is necessary to coordinate the research methods and developments among researchers, tourism professionals, and stakeholders both within Hungary and across Europe. Given that current tourism development strategies barely address climatic issues, future strategies should much more integrate the broad-ranging effects that climate change may pose to the tourism sector.

## Electronic supplementary material

Below is the link to the electronic supplementary material.

Supplementary Material 1

## Data Availability

The datasets on tourism climate indices generated and analysed during the current study are available from the corresponding author on reasonable request.

## References

[CR1] Adiguzel F, Bozdogan Sert E, Dinc Y et al (2022) Determining the relationships between climatic elements and thermal comfort and tourism activities using the tourism climate index for urban planning: a case study of Izmir Province. Theor Appl Climatol 147:1105–1120. 10.1007/s00704-021-03874-9

[CR2] Amelung B (2006) Global (environmental) change and tourism. Issues of scale and distribution. Dissertation, Universitaire Pers Maastricht, Amelung Publishers, Maastricht. 10.26481/dis.20060331sa

[CR3] Amelung B, Moreno A (2012) Costing the impact of climate change on tourism in Europe: results of the PESETA project. Clim Change 112:83–100. 10.1007/s10584-011-0341-0

[CR4] Amelung B, Viner D (2006) Mediterranean tourism: exploring the future with the Tourism Climatic Index. J Sustain Tour 14:349–366. 10.2167/jost549.0

[CR5] Aygün Oğur A (2023) Application of Holiday Climate Index (HCI): urban to potential alternative tourism attractions. Curr Urban Stud 11:497–520. 10.4236/cus.2023.113026

[CR6] Bafaluy D, Amengual A, Romero R, Homar V (2014) Present and future climate resources for various types of tourism in the Bay of Palma, Spain. Reg Environ Change 14:1995–2006. 10.1007/s10113-013-0450-6

[CR7] Bán B, Megyeri OA, Suga R (2021a) Az ALADIN5.2 és a REMO2015 regionális klímamodellek múltbeli időszakra vonatkozó eredményeinek validációja (Validation of ALADIN5.2 and REMO2015 regional climate models). KlimAdat (KEHOP-1.1.0) project report. https://www.met.hu/downloads.php?fn=/klimadat/doc/reports/KLIMADAT_beszamolo_ALADIN_REMO_final.pdf. Accessed 9 November 2024

[CR8] Bán B, Szépszó G, Allaga-Zsebeházi G, Somot S (2021b) ALADIN-Climate at the Hungarian Meteorological Service: from the beginnings to the present day’s results. Időjárás (Q J Hung Meteorol Serv) 125:647–673. 10.28974/idojaras.2021.4.6

[CR9] Bednar-Friedl B, Biesbroek R, Schmidt DN et al (2022) Europe. In: Pörtner H-O, Roberts DC, Tignor M et al (eds) Climate change 2022: impacts, adaptation and vulnerability. Contribution of working group II to the sixth assessment report of the Intergovernmental Panel on Climate Change. Cambridge University Press, Cambridge, pp 1817–1927. 10.1017/9781009325844.015

[CR10] Bihari Z, Lakatos M, Szentimrey T (2017) Felszíni megfigyelésekből készített rácsponti adatbázisok az Országos Meteorológiai Szolgálatnál (Gridded data series prepared from surface observation at Hungarian Meteorological Service). Légkör 62:148–151

[CR11] Bilgin B, Acar S, Demiralay Z, An N, Turp MT, Kurnaz ML (2024) A synthetic approach to the Holiday Climate Index for the Mediterranean Coast of Türkiye. Int J Biometeorol 68:1773–1787. 10.1007/s00484-024-02704-7 10.1007/s00484-024-02704-7 PMC11461600 38834880

[CR12] Burke M, Sipe N, Evans R, Mellifont D (2006) Climate, geography and the propensity to walk: environmental factors and walking trip rates in Brisbane. In: Proceedings of the 29th Australasian Transport Research Forum. Gold Coast, Queensland

[CR14] C3S (2024) Copernicus Climate Change Service (C3S), Climate Indicators for the European tourism sector. https://climate.copernicus.eu/tourism. Accessed 12 November 2024

[CR13] Carrillo J, González A, Pérez JC, Expósito FJ, Díaz JP (2022) Projected impacts of climate change on tourism in the Canary Islands. Reg Environ Change 22:61. 10.1007/s10113-022-01880-9

[CR15] de Freitas CR (2003) Tourism climatology: evaluating environmental information for decision making and business planning in the recreation and tourism sector. Int J Biometeorol 48:45–54. 10.1007/s00484-003-0177-z 12739109 10.1007/s00484-003-0177-z

[CR16] de Freitas CR, Scott D, McBoyle G (2008) A second generation climate index for tourism (CIT): specification and verification. Int J Biometeorol 52:399–407. 10.1007/s00484-007-0134-3 18097690 10.1007/s00484-007-0134-3

[CR17] Demiroglu OC, Saygili-Araci FS, Pacal A, Hall CM, Kurnaz ML (2020) Future Holiday Climate Index (HCI) performance of urban and beach destinations in the Mediterranean. Atmosphere 11:911. 10.3390/atmos11090911

[CR18] Dubois G, Ceron JP, Dubois C, Frias MD, Herrera S (2016) Reliability and usability of tourism climate indices. Earth Perspect 3:2. 10.1186/s40322-016-0034-y

[CR19] Fagence M, Kevan S (1998) Migration, recreation and tourism: human responses to climate differences. In: Auliciems A, de Dear R, Fagence M, Kolkstein LS, Kevan SD, Szokolay SV, Webb AR (eds) Advances in bioclimatology 5. Springer-Verlag, Berlin, Heidelberg, pp 133–160. 10.1007/978-3-642-80419-9_6

[CR20] Gómez Martín MB (2005) Weather, climate and tourism. A geographical perspective. Ann Tour Res 32:571–591. 10.1016/j.annals.2004.08.004

[CR21] Gössling S, Bredberg M, Randow A, Sandström E, Svensson P (2006) Tourist perceptions of climate change: A study of international tourists in Zanzibar. Curr Issues Tour 9:419–435. 10.2167/cit265.0

[CR22] Gössling S, Scott D, Hall CM, Ceron J-P, Dubois G (2012) Consumer behaviour and demand response of tourists to climate change. Ann Tour Res 39:36–58. 10.1016/j.annals.2011.11.002

[CR23] Hamilton JM, Lau MA (2005) The role of climate information in tourist destination choice decision-making. In: Gössling S, Hall CM (eds) Tourism and global environmental change. Routledge, London, pp 229–250

[CR24] Hasanah NAI, Maryetnowati D, Edelweis FN, Indriyani F, Nugrahayu Q (2020) The climate comfort assessment for tourism purposes in Borobudur Temple Indonesia. Heliyon 6:e05828. 10.1016/j.heliyon.2020.e05828 33426335 10.1016/j.heliyon.2020.e05828 PMC7777063

[CR25] Hawkins E, Osborne TM, Ho CK, Challinor AJ (2013) Calibration and bias correction of climate projections for crop modelling: an idealised case study over Europe. Agric Meteorol 170:19–31. 10.1016/j.agrformet.2012.04.007

[CR26] HCSO (2023) Turizmus-szatellitszámlák, 2022 (Tourism Satellit Accounts, 2022). Hungarian Central Statistical Office, Budapest. https://www.ksh.hu/s/kiadvanyok/turizmus-szatellitszamlak-2022/turizmus-szatellitszamlak-2022.pdf. Accessed 17 June 2024

[CR27] HCSO (2024a) Number and duration of domestic trips with overnight stay by main motivation. Hungarian Central Statistical Office, Budapest. https://www.ksh.hu/stadat_files/tur/en/tur0015.html. Accessed 17 June 2024

[CR28] HCSO (2024b) The number of inbound trips to Hungary and the related expenditures by main motivation. Hungarian Central Statistical Office, Budapest. https://www.ksh.hu/stadat_files/tur/en/tur0005.html. Accessed 17 June 2024

[CR29] HCSO (2024c) Snapshots 2023, tourism, catering. Hungarian Central Statistical Office, Budapest. https://www.ksh.hu/s/snapshots-2023/#/kiadvany/tourism-catering. Accessed 29 October 2024

[CR30] HCSO (2024d) Summary tables, tourism, catering. Hungarian Central Statistical Office, Budapest. https://www.ksh.hu/stadat_eng?theme=tur. Accessed 29 October 2024

[CR31] Hein L, Metzger MJ, Moreno A (2009) Potential impacts of climate change on tourism; a case study for Spain. Curr Opin Environ Sustain 1:170–178. 10.1016/j.cosust.2009.10.011

[CR33] Höppe P (1999) The physiological equivalent temperature – a universal index for the biometeorological assessment of the thermal environment. Int J Biometeorol 43:71–75. 10.1007/s004840050118 10552310 10.1007/s004840050118

[CR32] Houghten FC, Yaglou CP (1923) Determining equal comfort lines. J Am Soc Heat Vent Eng 29:165–176

[CR34] Kántor N, Kovács A, Takács Á (2016) Seasonal differences in the subjective assessment of outdoor thermal conditions and the impact of analysis techniques on the obtained results. Int J Biometeorol 60:1615–1635. 10.1007/s00484-016-1151-x 27029381 10.1007/s00484-016-1151-x

[CR35] Kjellström E, Nikulin G, Strandberg G et al (2018) European climate change at global mean temperature increases of 1.5 and 2°C above pre-industrial conditions as simulated by the EURO-CORDEX regional climate models. Earth Syst Dyn 9:459–478. 10.5194/esd-9-459-2018

[CR36] Kovács A (2017) A turisztikai klímapotenciál értékelése eredeti, valamint továbbfejlesztett és a magyar lakossághoz adaptált eszközökkel (Evaluation of the tourism climate potential based on original and improved methods adapted to Hungarian population). Dissertation, University of Szeged. 10.14232/phd.3860

[CR37] Kovács A, Király A (2021) Assessment of climate change exposure of tourism in Hungary using observations and regional climate model data. Hung Geogr Bull 70:215–231. 10.15201/hungeobull.70.3.2

[CR38] Kovács A, Unger J (2014a) Modification of the Tourism Climatic Index to Central European climatic conditions – examples. Időjárás (Q J Hung Meteorol Serv) 118:147–166

[CR39] Kovács A, Unger J (2014b) Analysis of tourism climatic conditions in Hungary considering the subjective thermal sensation characteristics of the south-Hungarian residents. Acta Climatol Chorol Univ Szeged 47–48:77–84

[CR41] Kovács A, Unger J, Gál CV, Kántor N (2016) Adjustment of the thermal component of two tourism climatological assessment tools using thermal perception and preference surveys from Hungary. Theor Appl Climatol 125:113–130. 10.1007/s00704-015-1488-9

[CR40] Kovács A, Németh Á, Unger J, Kántor N (2017) Tourism climatic conditions of Hungary – present situation and assessment of future changes. Időjárás (Q J Hung Meteorol Serv) 121:79–99

[CR42] Ma S, Craig CA, Feng S (2020) The Camping Climate Index (CCI): the development, validation, and application of a camping-sector tourism climate index. Tour Manag 80:104105. 10.1016/j.tourman.2020.104105

[CR43] Ma S, Craig CA, Feng S, Liu C (2023) Climate resources at United States National Parks: a tourism climate index approach. Tour Recreat Res 48:710–724. 10.1080/02508281.2021.1946652

[CR44] Matthews L, Scott D, Andrey J (2021) Development of a data-driven weather index for beach parks tourism. Int J Biometeorol 65:749–762. 10.1007/s00484-019-01799-7 31522261 10.1007/s00484-019-01799-7

[CR45] Matzarakis A (2007) Assessment method for climate and tourism based on daily data. In: Matzarakis A, de Freitas CR, Scott D (eds) Developments in tourism climatology. International Society of Biometeorology, Freiburg, pp 52–58

[CR46] Matzarakis A, Rutz F, Mayer H (2010) Modelling radiation fluxes in simple and complex environments: basics of the RayMan model. Int J Biometeorol 54:131–139. 10.1007/s00484-009-0261-0 19756771 10.1007/s00484-009-0261-0

[CR47] Megyeri-Korotaj OA, Bán B, Suga R, Allaga-Zsebeházi G, Szépszó G (2023) Assessment of climate indices over the Carpathian Basin based on ALADIN5.2 and REMO2015 regional climate model simulations. Atmosphere 14:448. 10.3390/atmos14030448

[CR48] Mieczkowski ZT (1985) The tourism climatic index: a method of evaluating world climates for tourism. Can Geogr 29:220–233. 10.1111/j.1541-0064.1985.tb00365.x

[CR49] Moreno A (2010) Mediterranean tourism and climate (change): a survey based study. Tour Hosp Plan Dev 7:253–265. 10.1080/1479053X.2010.502384

[CR50] Morgan R, Gatell E, Junyent R, Micallef A, Özhan E, Williams AT (2000) An improved user-based beach climate index. J Coast Conserv 6:41–50. 10.1007/BF02730466

[CR51] Moss RH, Edmonds JA, Hibbard KA et al (2010) The next generation of scenarios for climate change research and assessment. Nature 463:747–756. 10.1038/nature08823 20148028 10.1038/nature08823

[CR52] Perch-Nielsen SL, Amelung B, Knutti R (2010) Future climate resources for tourism in Europe based on the daily Tourism Climatic Index. Clim Change 103:363–381. 10.1007/s10584-009-9772-2

[CR53] Perry AH (1997) Recreation and tourism. In: Thompson RD, Perry A (eds) Applied climatology: principles and practice. Routledge, London, pp 240–248

[CR54] Prinsloo AS, Fitchett JM (2024) Quantifying climatic suitability for tourism in Southwest Indian Ocean Tropical Islands: applying the Holiday Climate Index to Réunion Island. Int J Biometeorol 68:1717–1728. 10.1007/s00484-024-02700-x 10.1007/s00484-024-02700-x PMC11461609 38744706

[CR55] Rutty M, Scott D (2014) Thermal range of coastal tourism resort microclimates. Tour Geogr 16:346–363. 10.1080/14616688.2014.932833

[CR56] Rutty M, Scott D, Matthews L, Burrowes R, Trotman A, Mahon R, Charles A (2020) An inter-comparison of the Holiday Climate Index (HCI:Beach) and the Tourism Climate Index (TCI) to explain Canadian tourism arrivals to the Caribbean. Atmosphere 11:412. 10.3390/atmos11040412

[CR57] Samarasinghe JT, Wickramarachchi CP, Makumbura RK, Meddage P, Gunathilake MB, Muttil N, Rathnayake U (2023) Performances of Holiday Climate Index (HCI) for urban and beach destinations in Sri Lanka under changing climate. Climate 11:48. 10.3390/cli11030048

[CR58] Scott D, Lemieux C (2010) Weather and climate information for tourism. Proced Environ Sci 1:146–183. 10.1016/j.proenv.2010.09.011

[CR59] Scott D, McBoyle G (2001) Using a ‘tourism climate index’ to examine the implications of climate change for climate as a tourism resource. In: Matzarakis A, de Freitas CR (eds) Proceedings of the First International Workshop on Climate, Tourism and Recreation. International Society of Biometeorology, Halkidiki, pp 69–88

[CR60] Scott D, McBoyle G, Schwartzentruber M (2004) Climate change and the distribution of climatic resources for tourism in North America. Clim Res 27:105–117. 10.3354/cr027105

[CR61] Scott D, Hall CM, Gössling S (2012) Tourism and climate change: impacts, adaptation and mitigation. Routledge, Abingdon

[CR62] Scott D, Rutty M, Amelung B, Tang M (2016) An inter-comparison of the Holiday Climate Index (HCI) and the Tourism Climate Index (TCI) in Europe. Atmosphere 7:80. 10.3390/atmos7060080

[CR63] Smith K (1993) The influence of weather and climate on recreation and tourism. Weather 48:398–404. 10.1002/j.1477-8696.1993.tb05828.x

[CR64] Suga R, Megyeri-Korotaj OA, Allaga-Zsebeházi G (2021) Sensitivity study of the REMO regional climate model to domain size. Adv Sci Res 18:157–167. 10.5194/asr-18-157-2021

[CR65] Sütő A, Fejes L (2019) A turizmus ágazat jelenlegi és potenciális éghajlati sérülékenységének területi különbségei Magyarországon (Territorial differences in existing and potential climate vulnerability of the tourism sector in Hungary). Tér és Társad 33:108–126. 10.17649/TET.33.3.3178

[CR66] TPCC (2023) Tourism and climate change stocktake 2023. Tourism Panel on Climate Change. https://tpcc.info/stocktake-report/. Accessed 26 June 2024

[CR67] UNWTO (2008) Climate change and tourism – Responding to global challenges. World Tourism Organization and United Nations Environment Programme, Madrid

[CR68] Velea L, Gallo A, Bojariu R, Irimescu A, Craciunescu V, Puiu S (2022) Holiday Climate Index: Urban – Application for urban and rural areas in Romania. Atmosphere 13:1519. 10.3390/atmos13091519

[CR69] Velea L, Bojariu R, Irimescu A, Craciunescu V, Puiu S, Gallo A (2023) Climate suitability for tourism in Romania based on HCI: urban climate index in the near-future climate. Atmosphere 14:1020. 10.3390/atmos14061020

[CR70] Whittlesea E, Amelung B (2010) Cost-a South West: what could tomorrow’s weather and climate look like for tourism in the South West of England? National case study. South West Tourism, Exeter, Devon

[CR71] Yu DD, Rutty M, Scott D, Li S (2021) A comparison of the holiday climate index:beach and the tourism climate index across coastal destinations in China. Int J Biometeorol 65:741–748. 10.1007/s00484-020-01979-w 32761265 10.1007/s00484-020-01979-w PMC7406217

