# Supplementary material for: Projected climate suitability for Hungarian tourism in the 21st century: application of the Holiday Climate Index and modified Tourism Climate Index
Source: Int J Biometeorol. 2025 Apr 16;69(6):1429–42. doi: 10.1007/s00484-025-02901-y (PMC12141134; doi:10.1007/s00484-025-02901-y)
Supplement: Supplementary file 1 — Supplementary Material 1 [file 484_2025_2901_MOESM1_ESM.docx]

**Supplementary files**

**Article title:** Projected Climate Suitability for Hungarian Tourism in the 21st Century: Application of the Holiday Climate Index and Modified Tourism Climate Index

**Journal:** International Journal of Biometeorology

**Authors:** Attila Kovács^1*^, Gergely Molnár, Otília A. Megyeri-Korotaj

^1^Department of Atmospheric and Geospatial Data Sciences, University of Szeged, 2 Egyetem Str, HU-6722 Szeged, Hungary.

*Corresponding author e-mail address: kovacs.attila.05@szte.hu

**Table S1** Some basic aspects of tourism in Hungary for 2023 (data collection from HCSO 2024c, d)

| **Number of tourist accommodation bed-places on 31 July 2023 (thousands)** | 565 |
| --- | --- |
| **Number of tourism nights spent in tourist accommodation establishments (millions)** | 42 |
| **Number of tourism nights spent in tourist accommodation by top three months (millions)** | 1) August: 6,7  2) July: 6,5  3) June: 4,0 |
| **Number of tourism nights spent by foreign visitors in tourist accommodation by top three sending countries (millions)** | 1) Germany: 2,5  2) Czechia: 1,4  3) United Kingdom: 1,3 |
| **Top five Hungarian cities by number of tourism nights spent in tourist accommodation (millions)** | 1) Budapest: 14,162  2) Siófok: 1,117  3) Hajdúszoboszló: 1,112  4) Hévíz: 1,069  5) Balatonfüred: 0,888 |
| **Number of domestic same-day trips and tourist trips with overnight stay (millions)** | Same-day trips: 31,7  2–4 day trips: 9,6  5 and more day trips: 3,3 |
| **Number of inbound trips to Hungary by number of days (millions)** | Same-day trips: 36,5  2–4 day trips: 6,6  5 and more day trips: 6,3 |
| **Time spent on inbound trips to Hungary (million days)** | Same-day trips: 36,5  2–4 day trips: 20,2  5 and more day trips: 57,5 |
| **Number of inbound trips to Hungary by mode of transport (millions)** | Air: 5,2  Road: 44,2 |
| **Distribution of inbound overnight trips to Hungary by top three Hungarian regions (%) (the asked persons could indicate more than one region)** | 1) Budapest and Central Danube: 52,6  2) Western Transdanubia: 18,1  3) Lake Balaton: 13,3 |

**Table S2** The sub-indices and their basic meteorological input parameters, the rating score ranges of sub-indices and their weights utilised in TCI index (based on Mieczkowski 1985)

| **Basic meteorological parameters (monthly values)** | | **TCI sub-indices** | **Rating scores** | **Weight** |
| --- | --- | --- | --- | --- |
| daily maximum air temperature (°C)  daily minimum relative humidity (%) | daytime effective temperature (ET) (°C) | *CId*: daytime comfort index | –3 – 5 | 40% |
| daily mean air temperature (°C)  daily mean relative humidity (%) | daily effective temperature (ET) (°C) | *CIa*: daily comfort index | –3 – 5 | 10% |
| daily precipitation sum (mm) | | *P*: precipitation index | 0 – 5 | 20% |
| daily sunshine duration (hour) | | *S*: sunshine duration index | 0 – 5 | 20% |
| daily mean wind speed (km/h) | | *W*: wind speed index | 0 – 5 | 10% |

**Table S3** The sub-indices and their basic meteorological input parameters, the rating score ranges of sub-indices and their weights utilised in mTCI index (based on Kovács et al. 2016, 2017)

| **Basic meteorological parameters (daily values)** | | **mTCI sub-indices** | **Rating scores** | **Weight** |
| --- | --- | --- | --- | --- |
| daily maximum air temperature (°C)  daily minimum relative humidity (%)  daily mean wind speed (m/s)  daily mean cloud cover (octa) or global radiation (W/m^2^) | daily maximum Physiologically Equivalent Temperature (PET) (°C) | *CId*: daytime comfort index | 0 – 5 | 40% |
| daily mean air temperature (°C)  daily mean relative humidity (%)  daily mean wind speed (m/s)  daily mean cloud cover (octa) or global radiation (W/m^2^) | daily mean Physiologically Equivalent Temperature (PET) (°C) | *CIa*: daily comfort index | 0 – 5 | 10% |
| daily precipitation sum (mm) | | *P*: precipitation index | 0 – 5 | 20% |
| daily sunshine duration (hour) | | *S*: sunshine duration index | 0 – 5 | 20% |
| daily mean wind speed (km/h) | | *W*: wind speed index | 0 – 5 | 10% |

**Table S4** The sub-indices and their basic meteorological input parameters, the rating score ranges of sub-indices and their weights utilised in HCI index (based on Scott et al. 2016)

| **Basic meteorological parameters (daily values)** | | **HCI sub-indices** | **Rating scores** | **Weight** |
| --- | --- | --- | --- | --- |
| daily maximum air temperature (°C)  daily mean relative humidity (%) | Effective temperature (ET) or Humidex (°C) | *TC*: thermal comfort index | 0 – 10 | 40% |
| daily mean cloud cover (%) | | *A*: aesthetic index | 1 – 10 | 20% |
| daily precipitation sum (mm) | | *P*: physical (precipitation) index | –1 – 10 | 30% |
| daily mean wind speed (km/h) | | *W*: physical  (wind speed) index | –10 – 10 | 10% |

**Table S5** The overall rating systems of TCI (Mieczkowski 1985), mTCI (Mieczkowski 1985; Kovács et al. 2016, 2017) and HCI (Scott et al. 2016)

| **TCI and mTCI score** | | **HCI score** | |
| --- | --- | --- | --- |
| **Score** | **Descriptive rates** | **Score** | **Descriptive rates** |
| 90 – 100 | ideal | 90 – 100 | ideal |
| 80 – 90 | excellent | 80 – 90 | excellent |
| 70 – 80 | very good | 70 – 80 | very good |
| 60 – 70 | good | 60 – 70 | good |
| 50 – 60 | acceptable | 50 – 60 | acceptable |
| 40 – 50 | marginal | 40 – 50 | marginal |
| 30 – 40 | unfavourable | 20 – 40 | unacceptable |
| 20 – 30 | very unfavourable | 0 – 20 | dangerous |
| 10 – 20 | extremely unfavourable |  | |
| <10 | impossible |  |  |

**Table S6** Details on regional climate models applied in the study

| **Regional climate model** | **Boundary global model** | **Emission scenario** | **Resolution** | **Reference**  **period** | **Evaluation**  **period** |
| --- | --- | --- | --- | --- | --- |
| REMO2015 | MPI-ESM-LR | RCP4.5  RCP8.5 | 0.1° | 1971–2000 | 2041–2070  2071–2100 |
| ALADIN5.2 | CNRM-CM5 | RCP4.5  RCP8.5 |  |  |  |


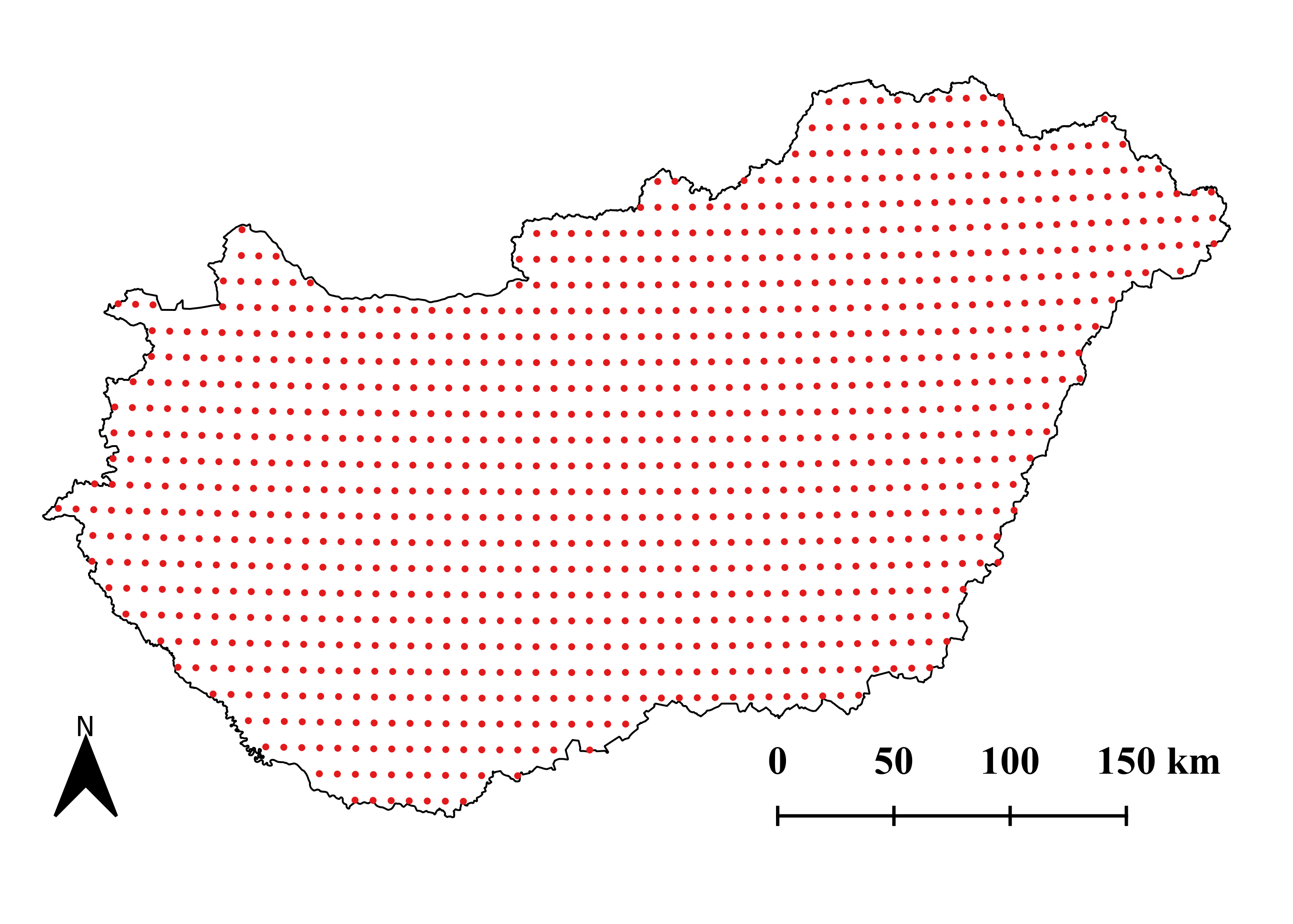


**Fig. S1** The grid system of observational database CarpatClim-HU and regional climate models REMO2015 and ALADIN5.2 (0.1° × 0.1° resolution)


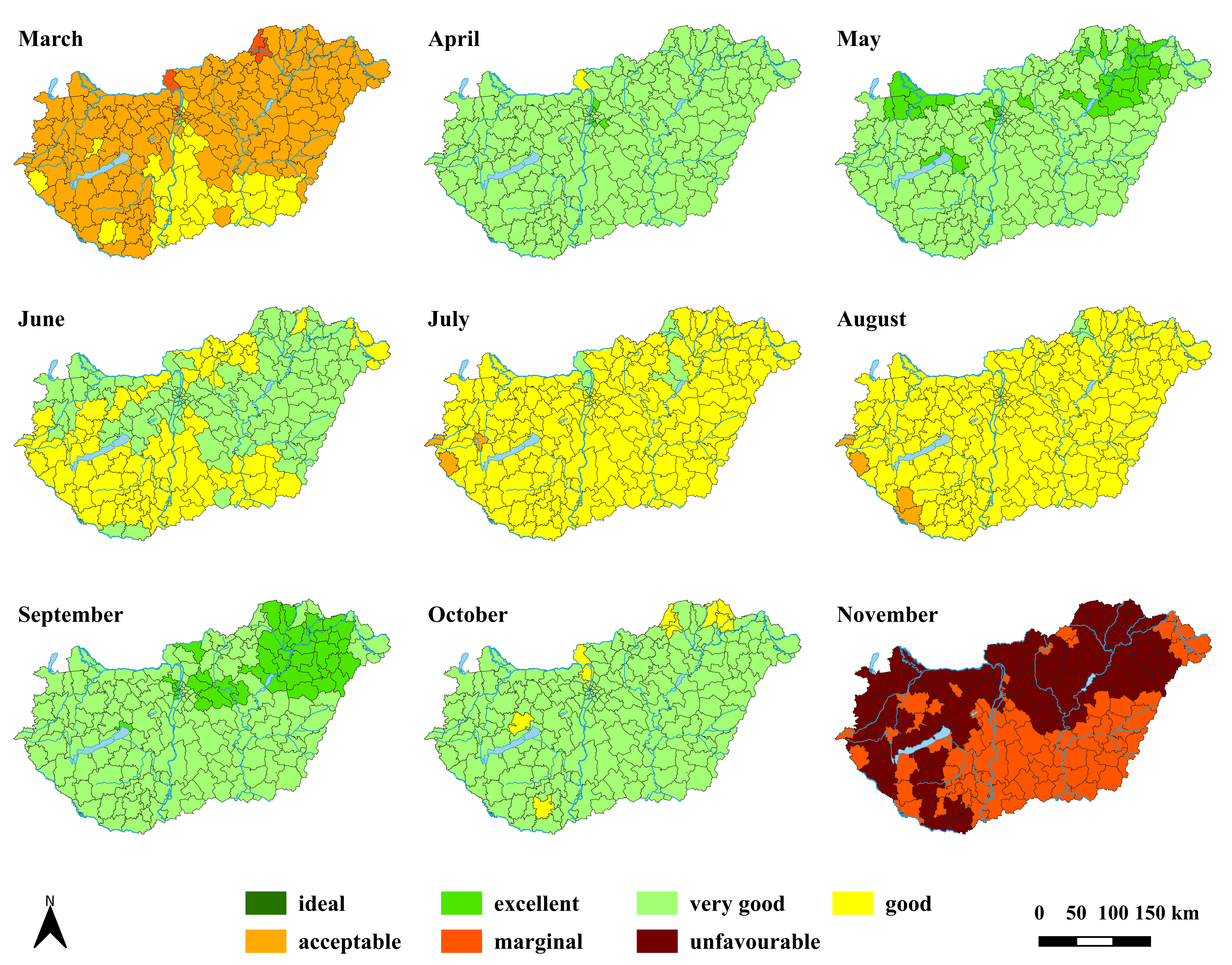


**Fig. S2** Spatial distribution of monthly mTCI ratings by district for the period 1971–2000 based on CarpatClim-HU observational database


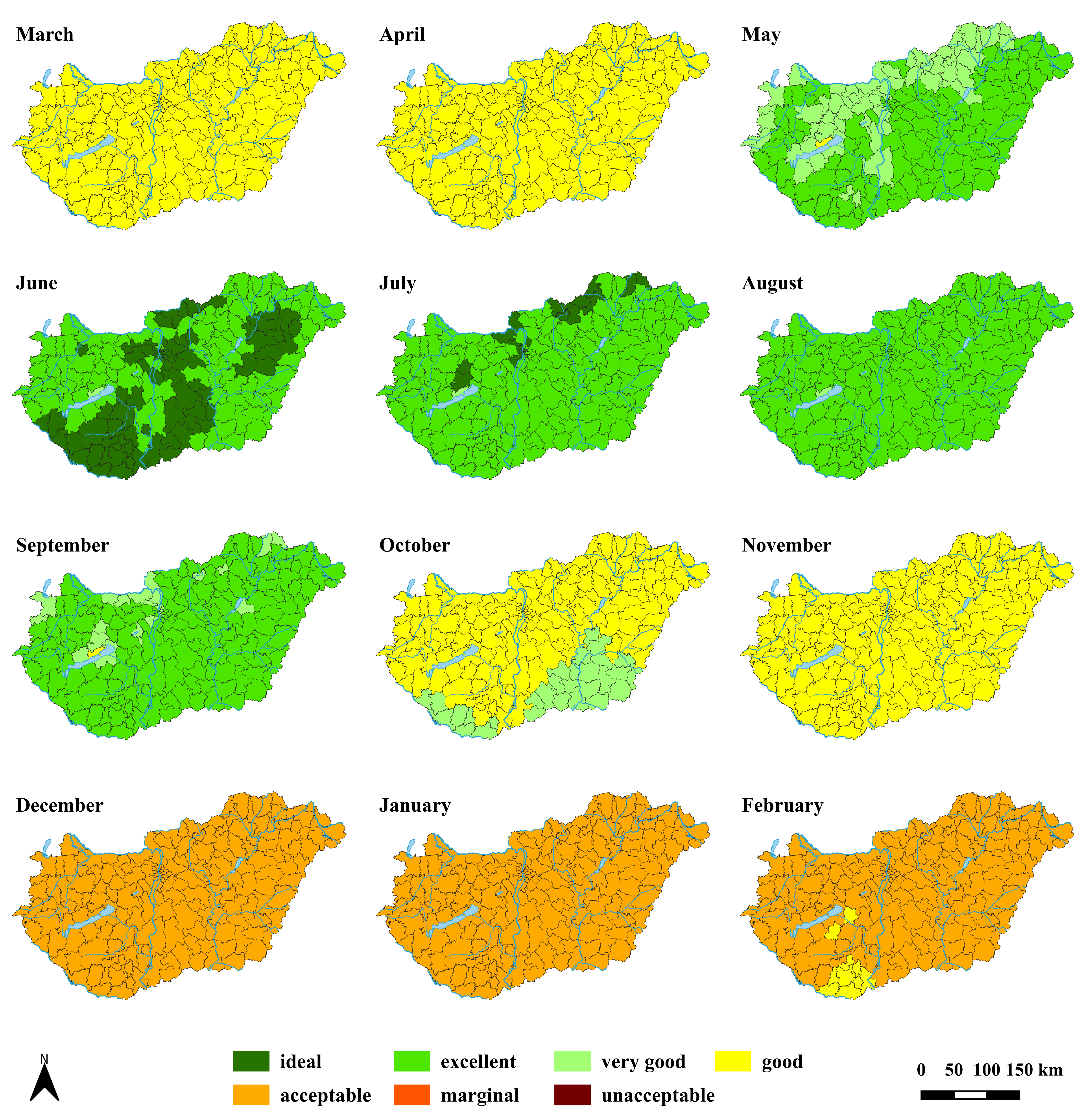


**Fig. S3** Spatial distribution of monthly HCI ratings by district for the period 1971–2000 based on REMO2015 simulation


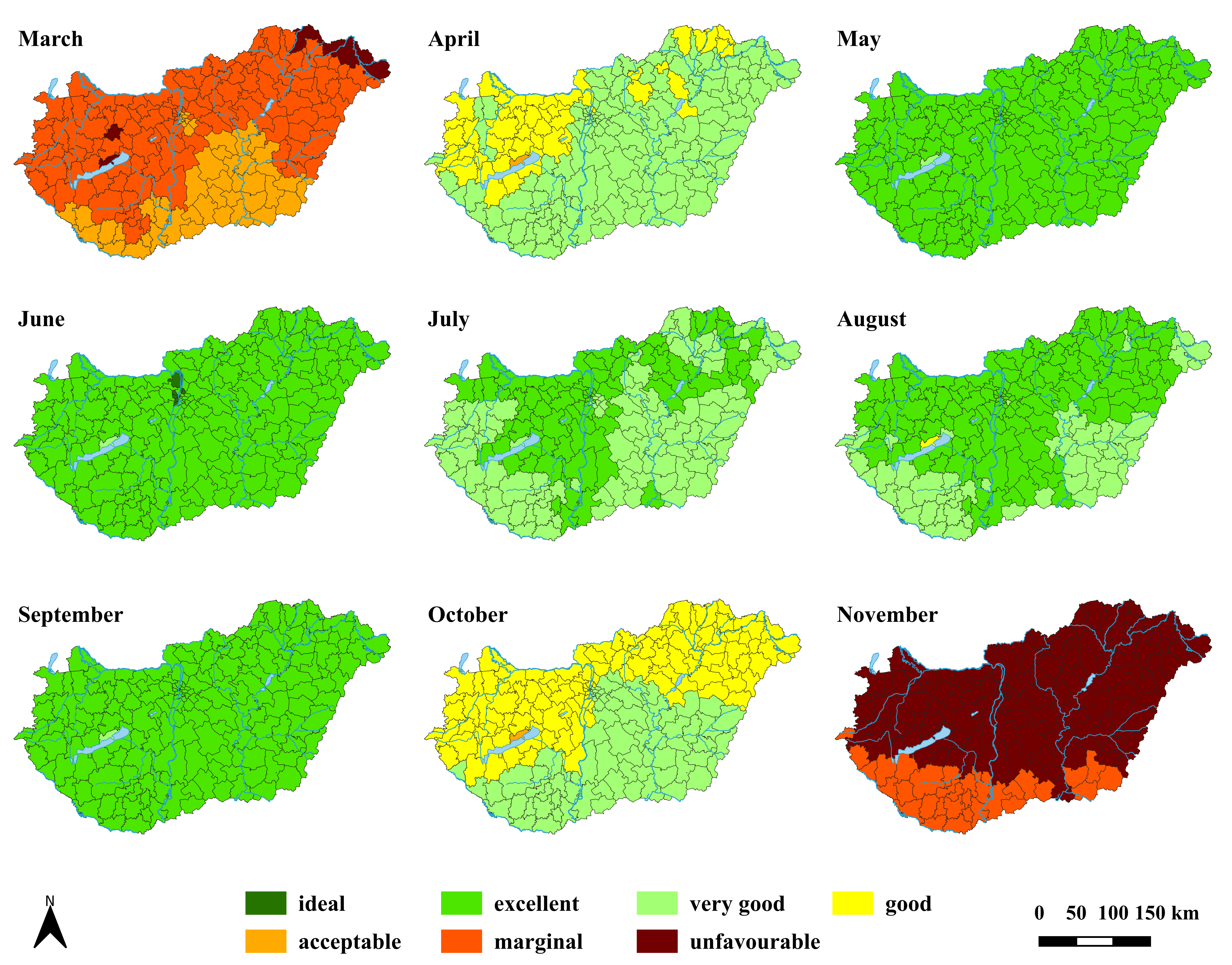


**Fig. S4** Spatial distribution of monthly mTCI ratings by district for the period 1971–2000 based on REMO2015 simulation


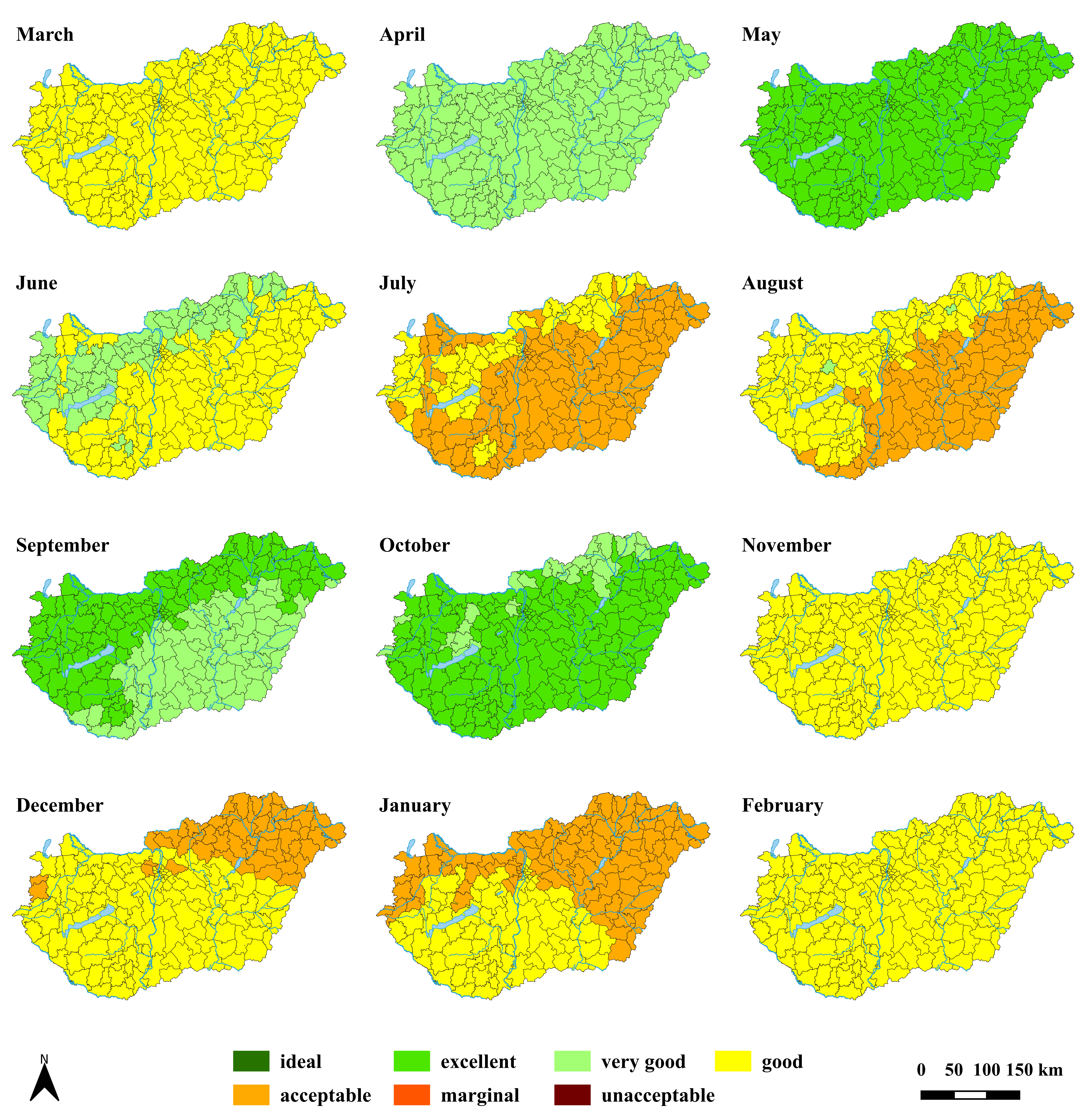


**Fig. S5** Spatial distribution of monthly HCI ratings by district for the period 1971–2000 based on ALADIN5.2 simulation


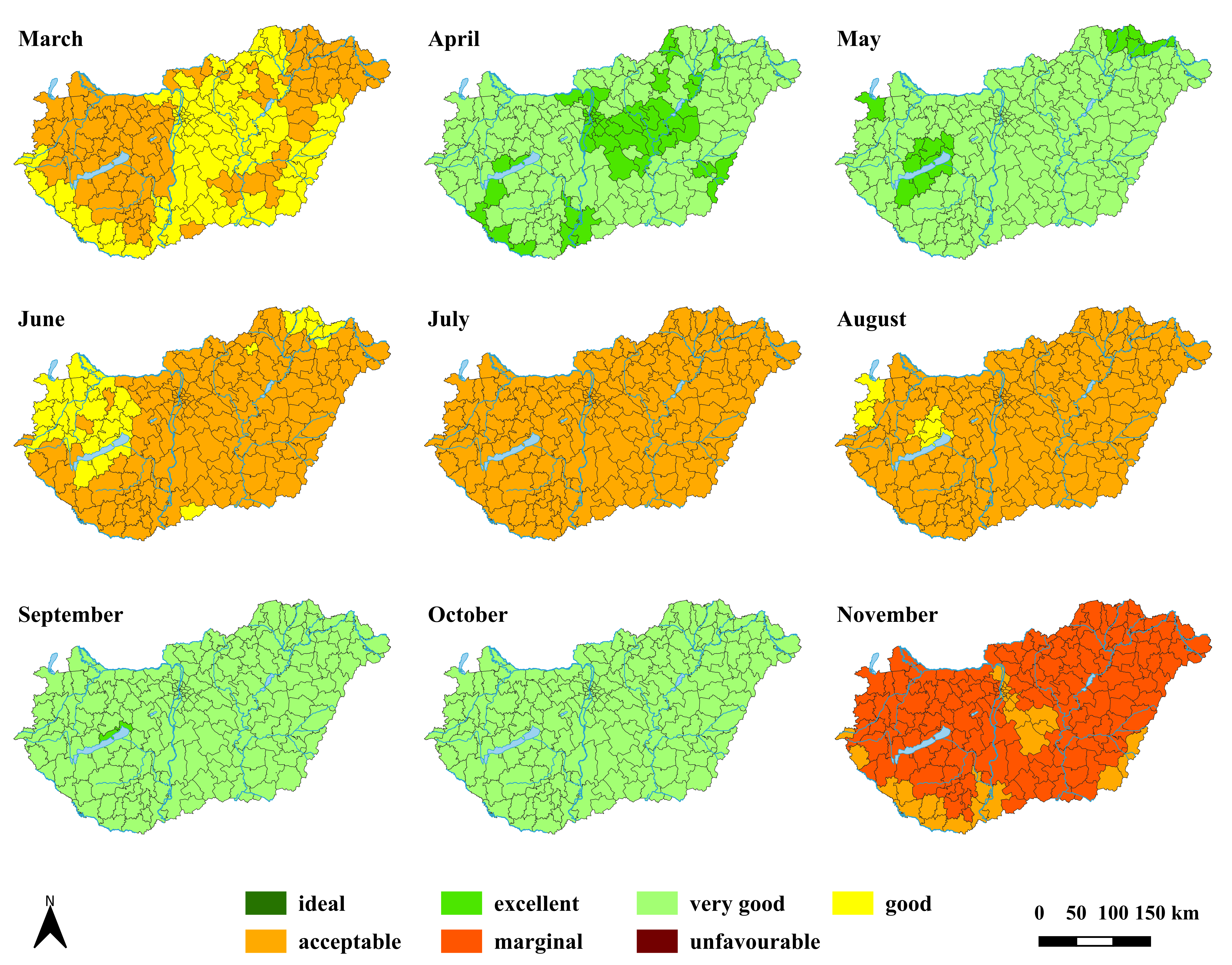


**Fig. S6** Spatial distribution of monthly mTCI ratings by district for the period 1971–2000 based on ALADIN5.2 simulation


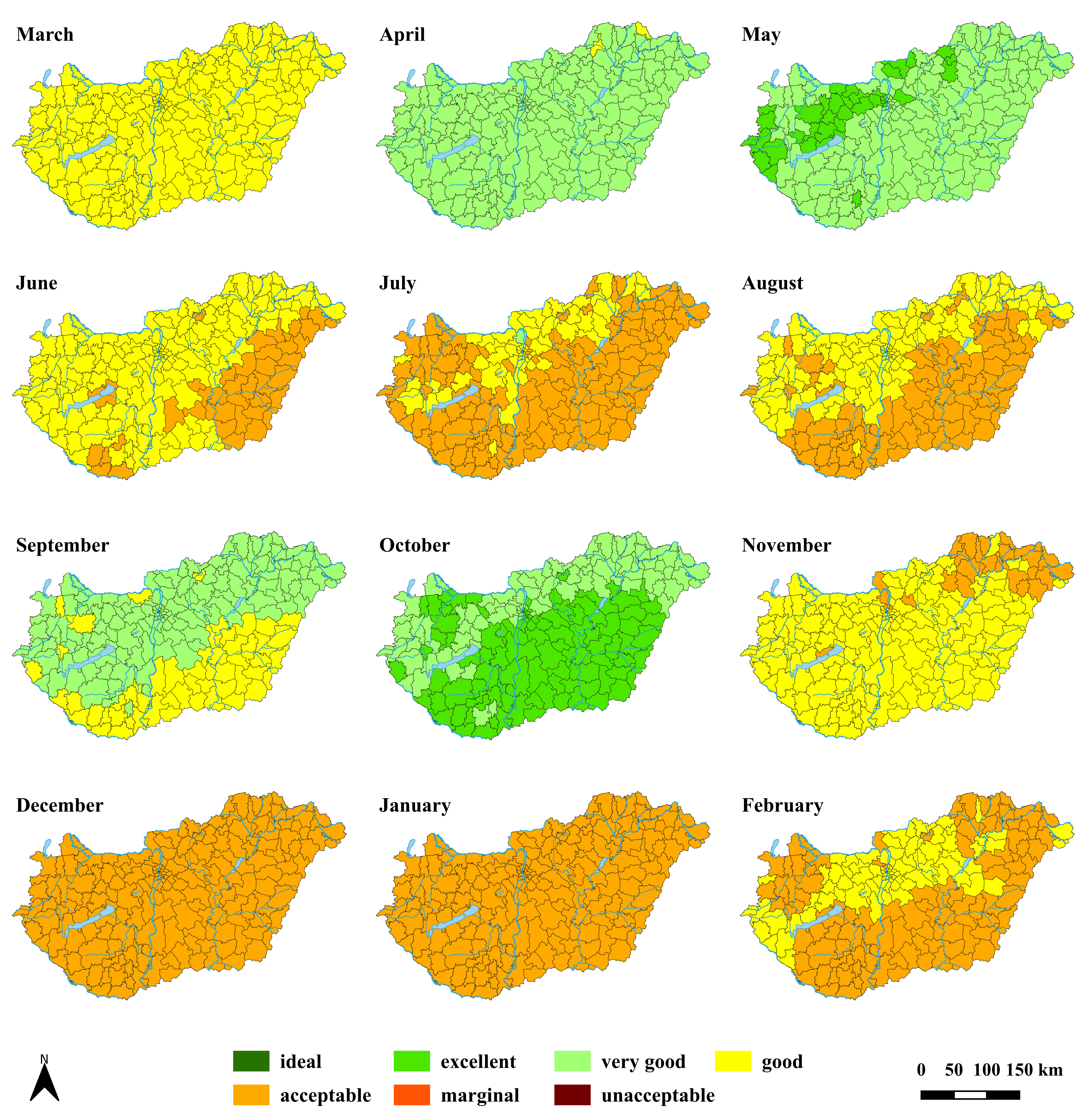


**Fig. S7** Spatial distribution of monthly HCI ratings by district for the period 2071–2100 based on the minimum values of simulated results


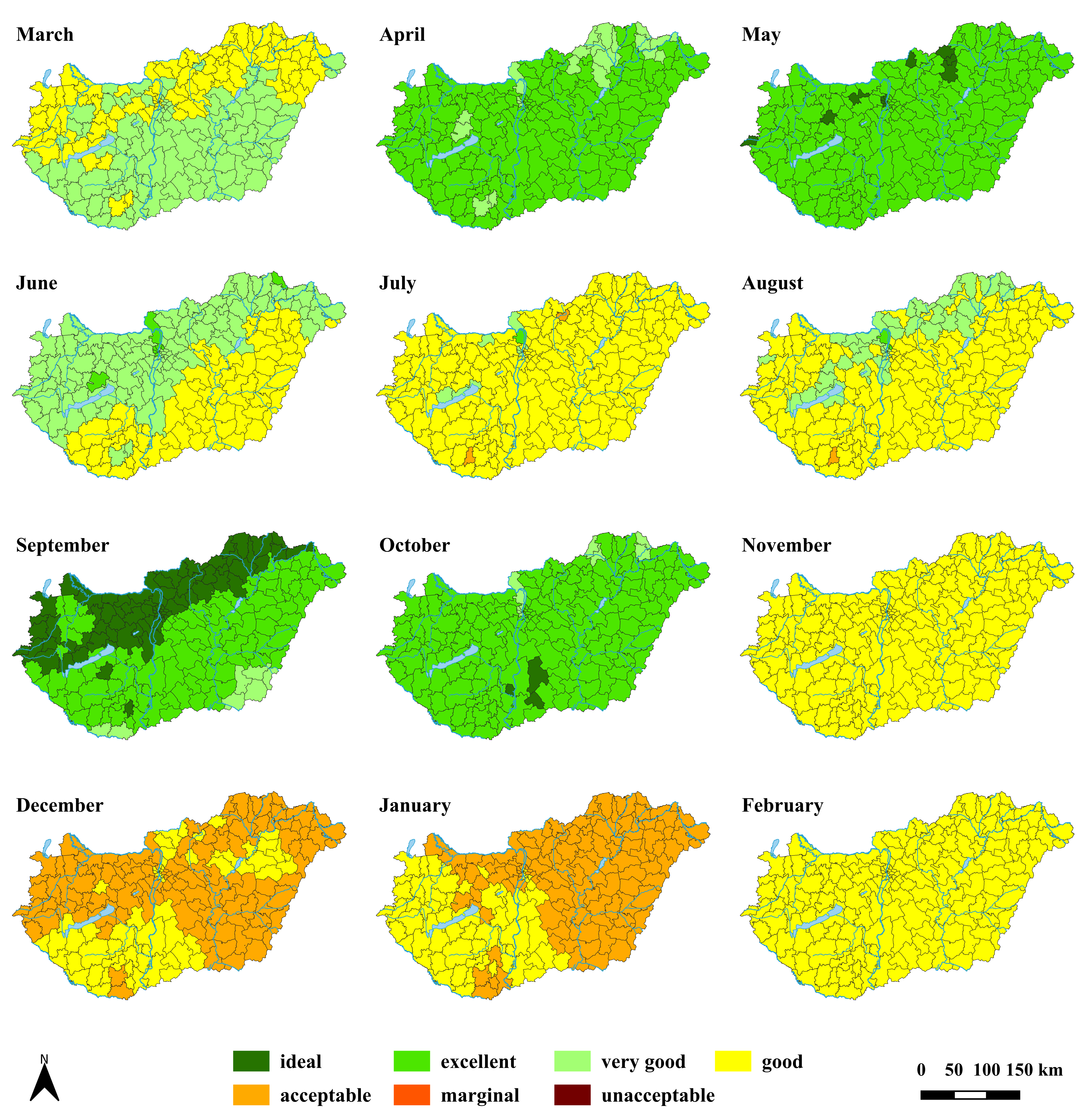


**Fig. S8** Spatial distribution of monthly HCI ratings by district for the period 2071–2100 based on the maximum values of simulated results


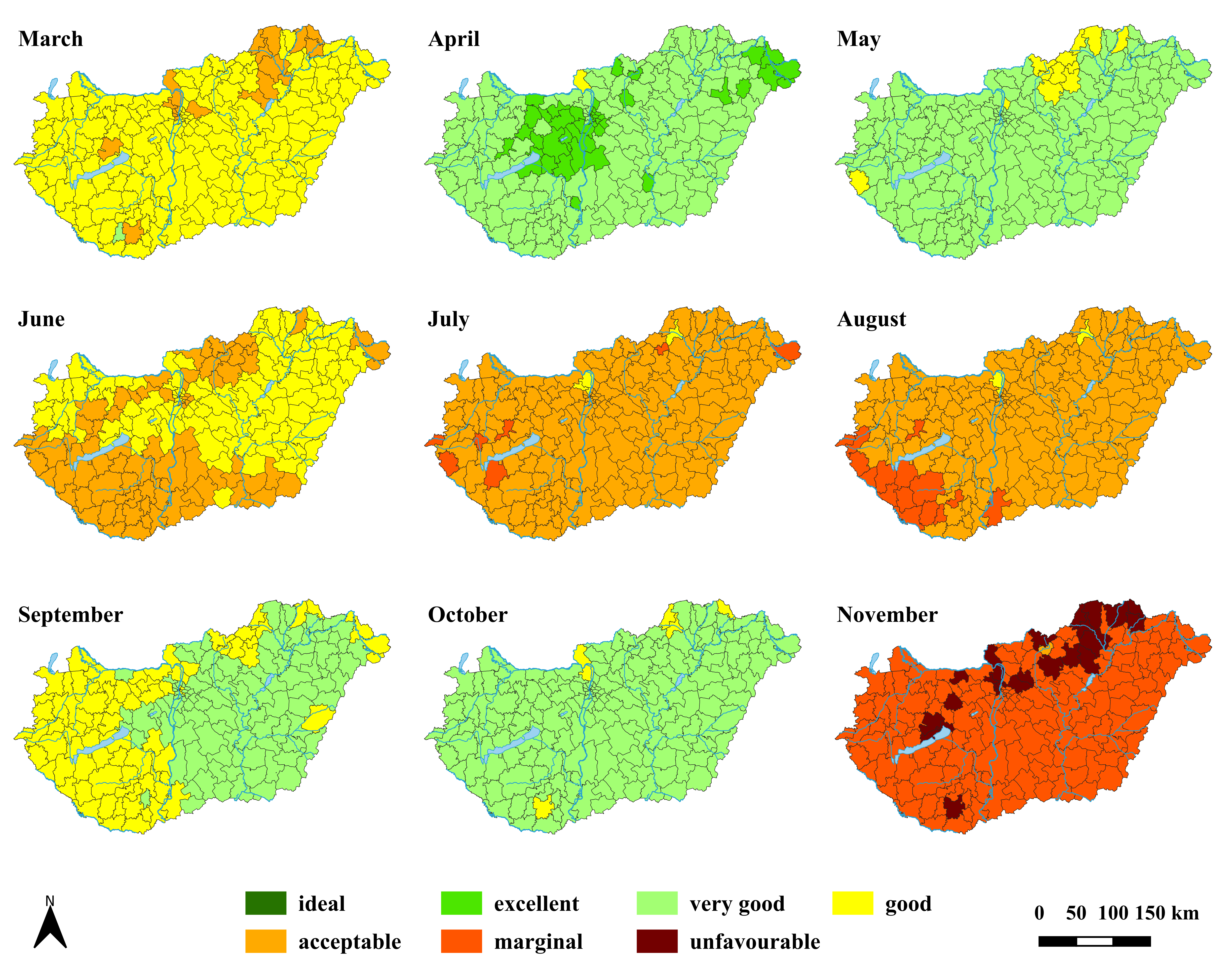


**Fig. S9** Spatial distribution of monthly mTCI ratings by district for the period 2071–2100 based on the minimum values of simulated results


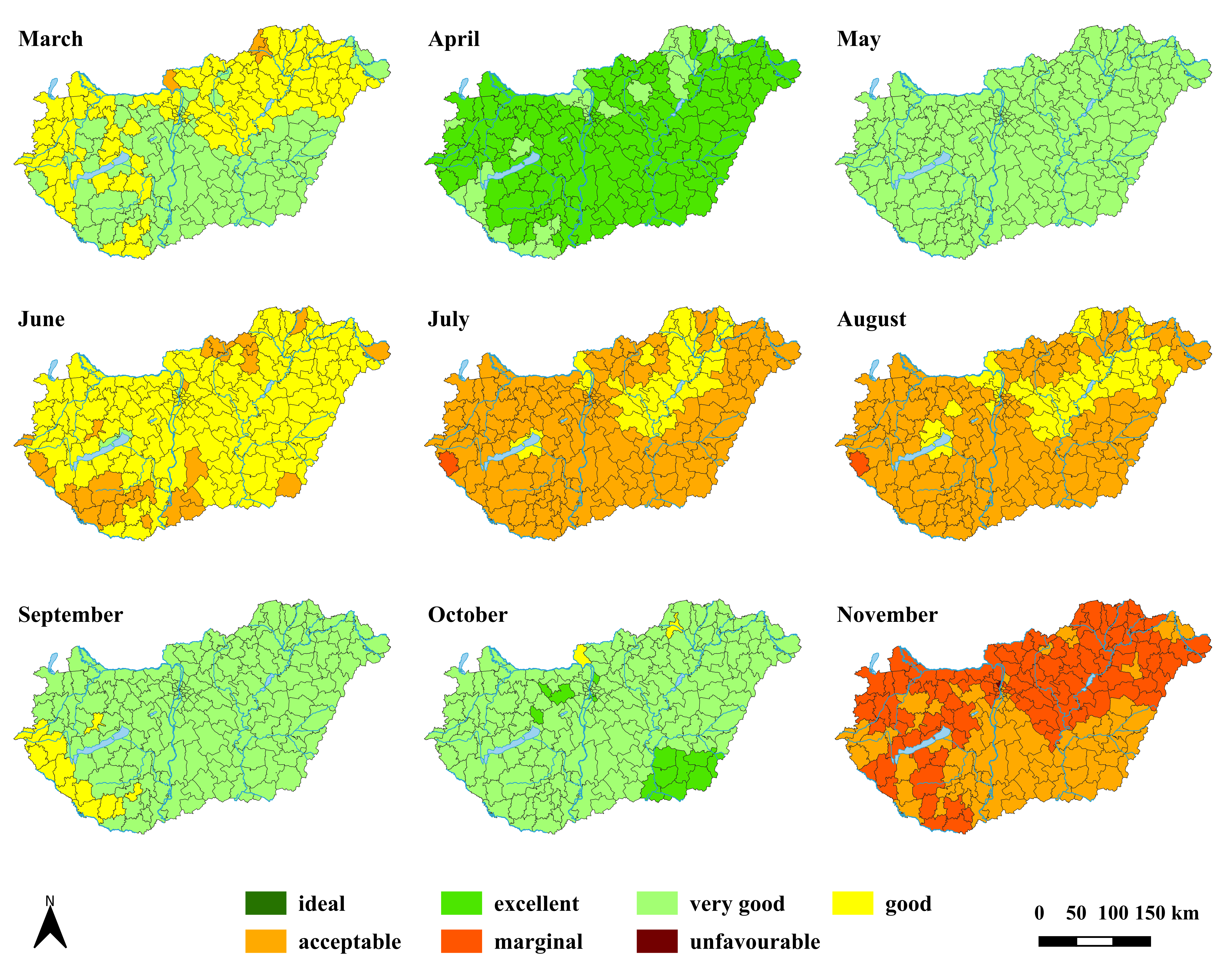


**Fig. S10** Spatial distribution of monthly mTCI ratings by district for the period 2071–2100 based on the median values of simulated results


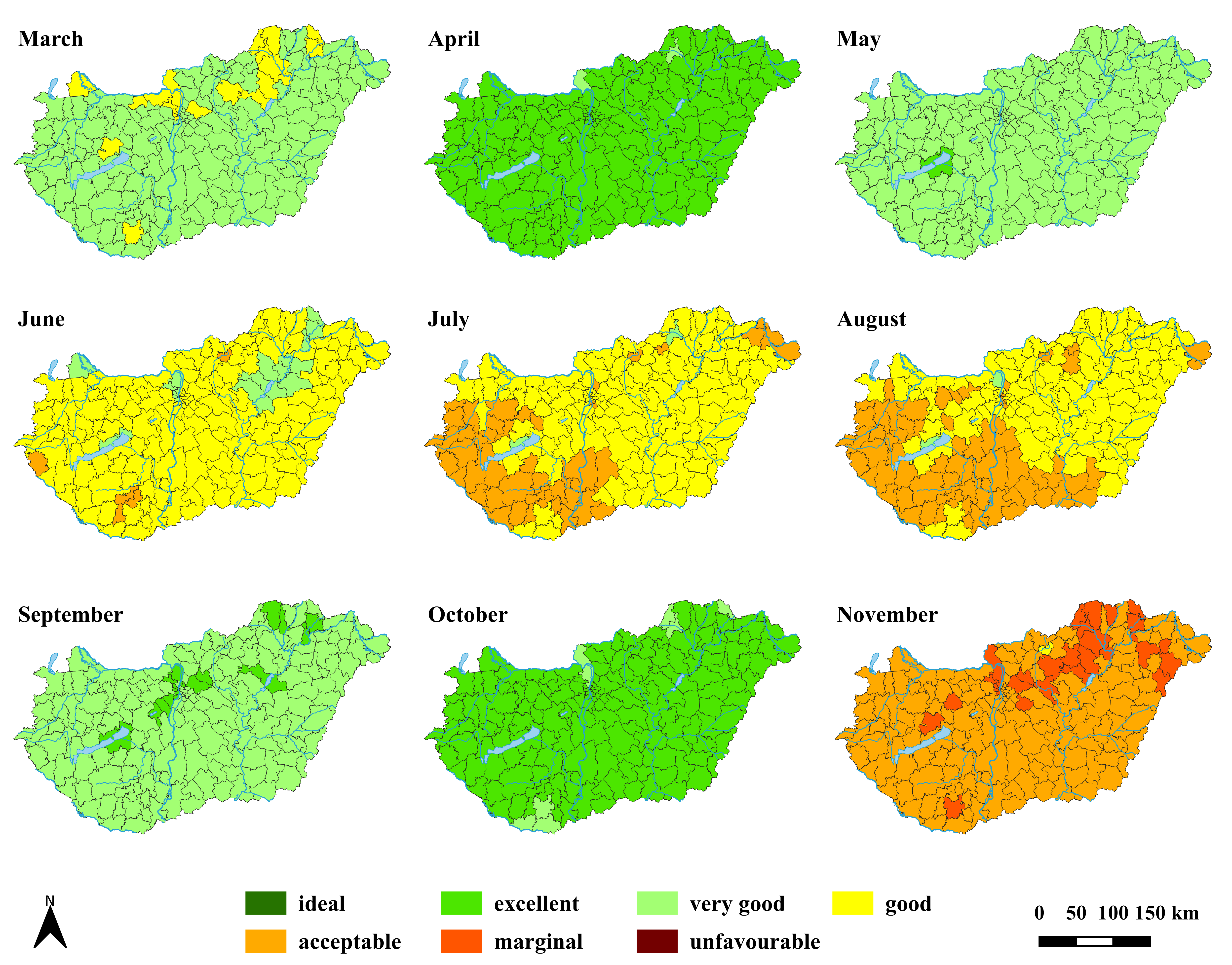


**Fig. S11** Spatial distribution of monthly mTCI ratings by district for the period 2071–2100 based on the maximum values of simulated results


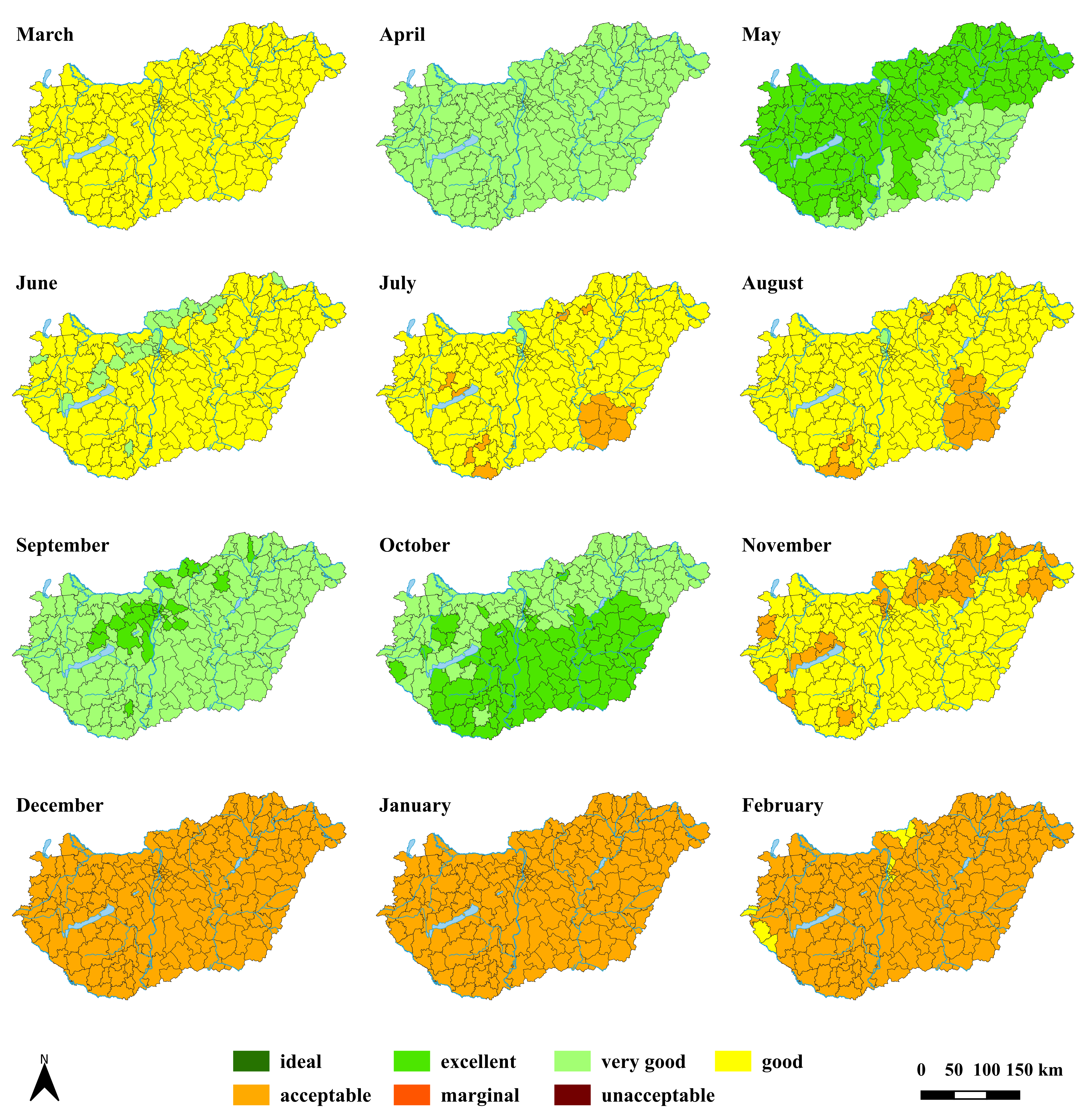


**Fig. S12** Spatial distribution of monthly HCI ratings by district for the period 2041–2070 based on the minimum values of simulated results


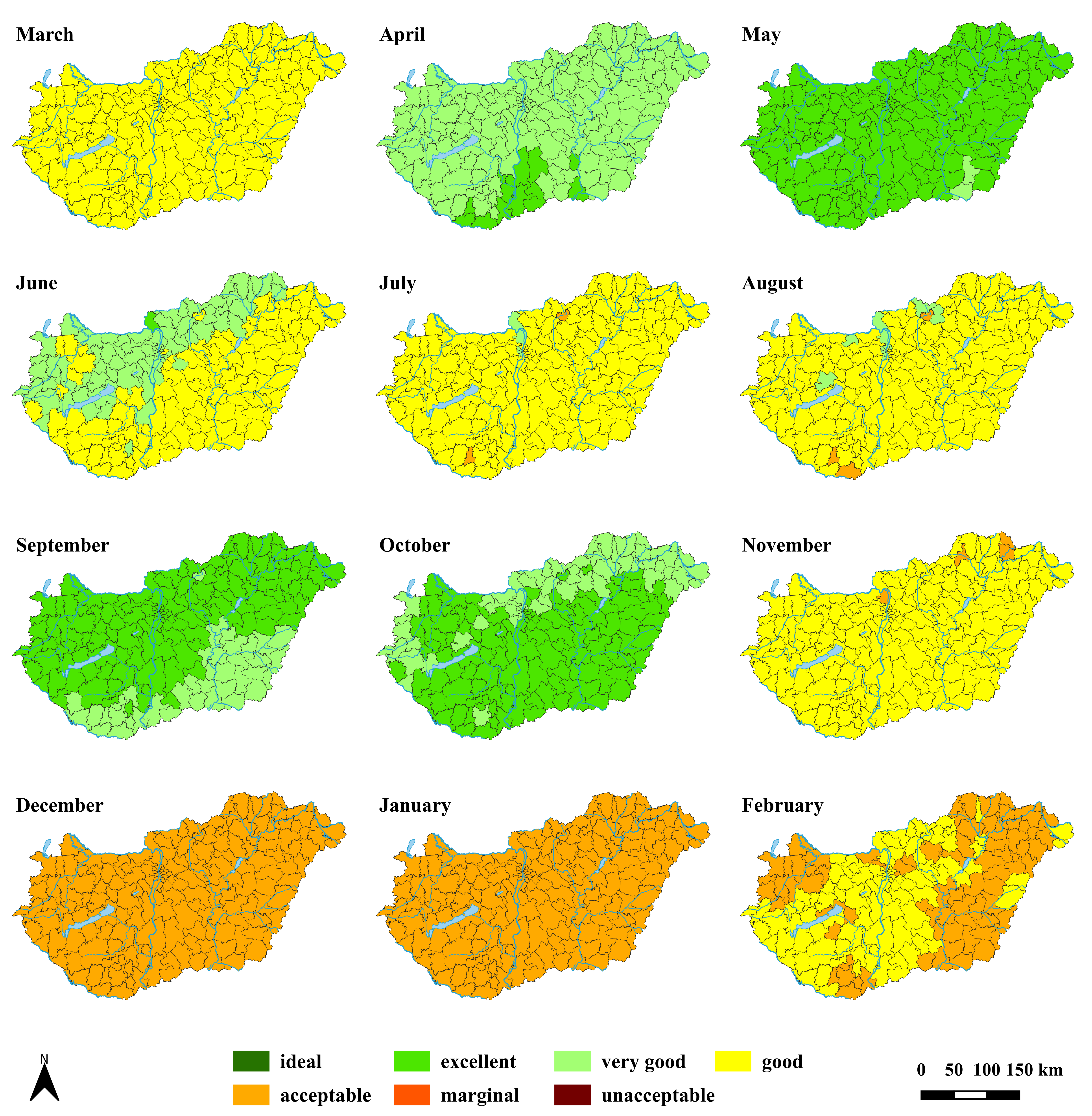


**Fig. S13** Spatial distribution of monthly HCI ratings by district for the period 2041–2070 based on the median values of simulated results


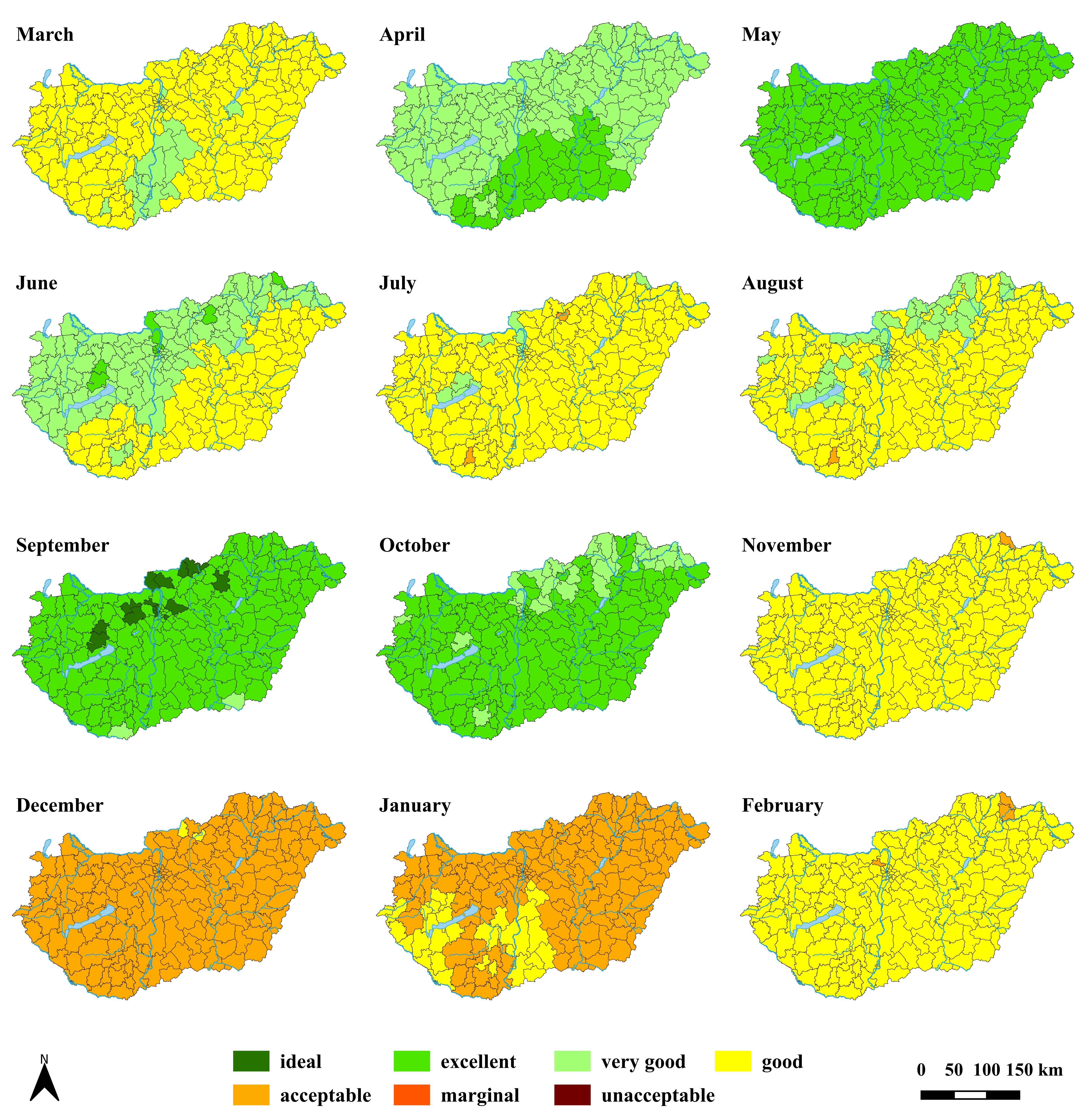


**Fig. S14** Spatial distribution of monthly HCI ratings by district for the period 2041–2070 based on the maximum values of simulated results


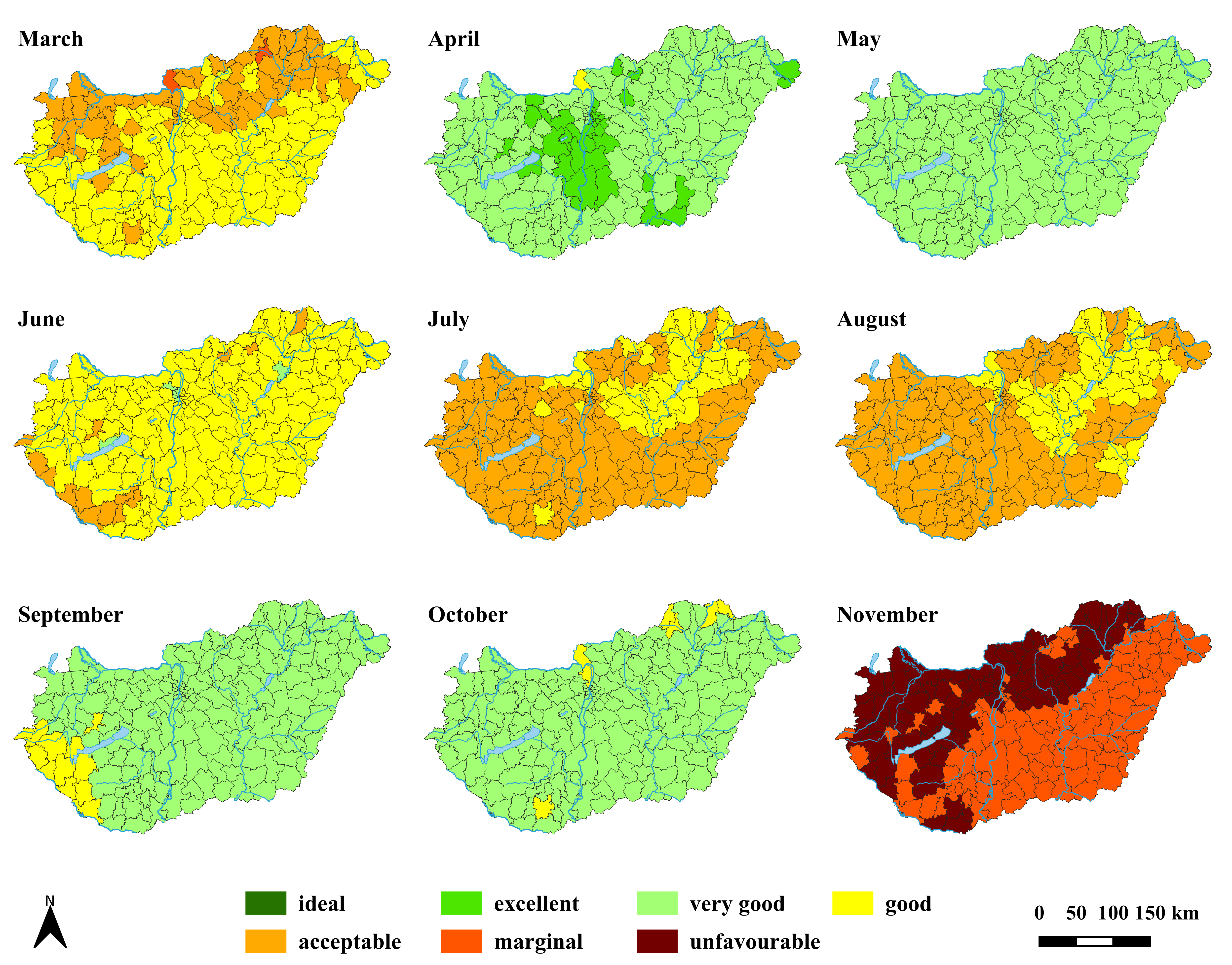


**Fig. S15** Spatial distribution of monthly mTCI ratings by district for the period 2041–2070 based on the minimum values of simulated results


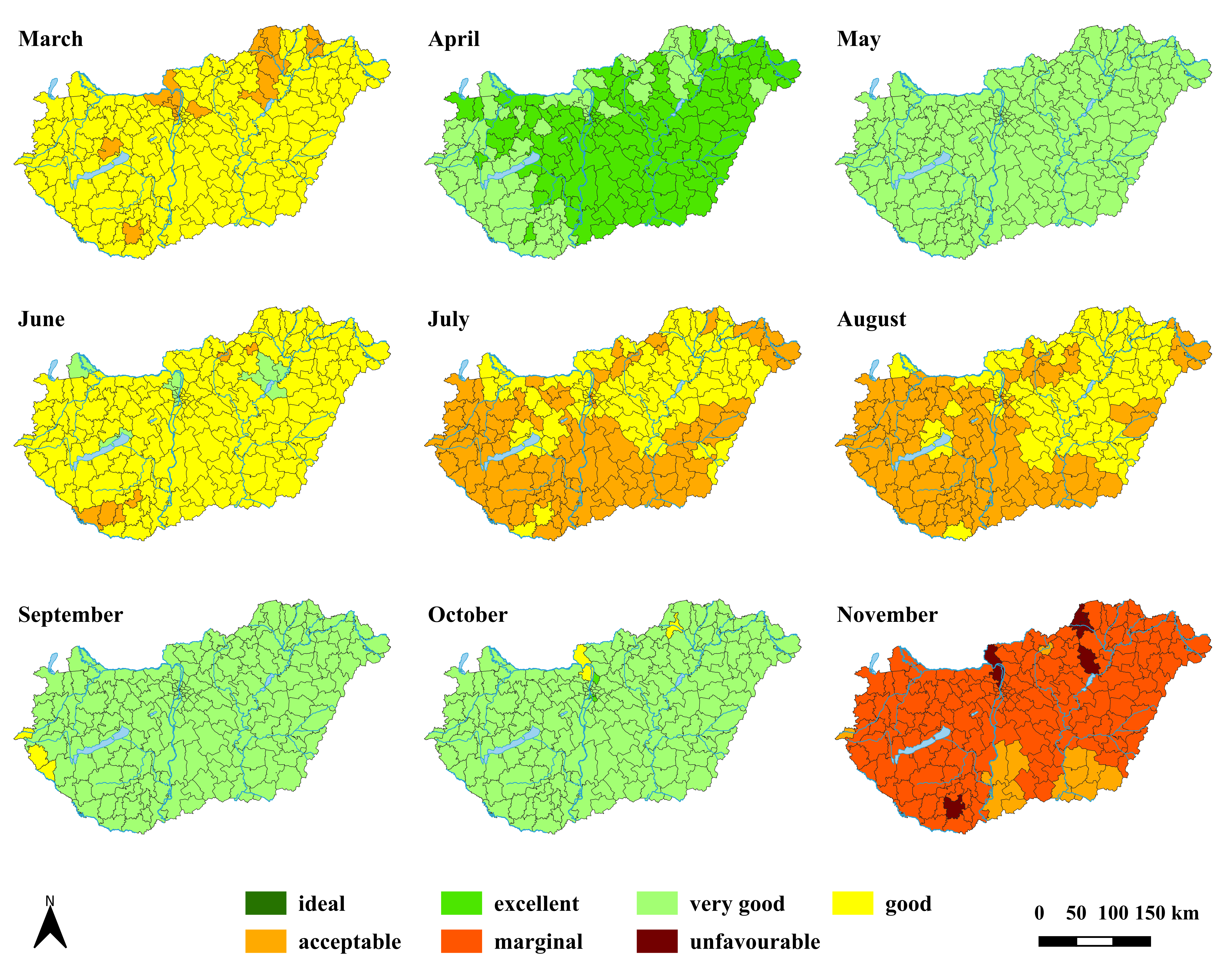


**Fig. S16** Spatial distribution of monthly mTCI ratings by district for the period 2041–2070 based on the median values of simulated results


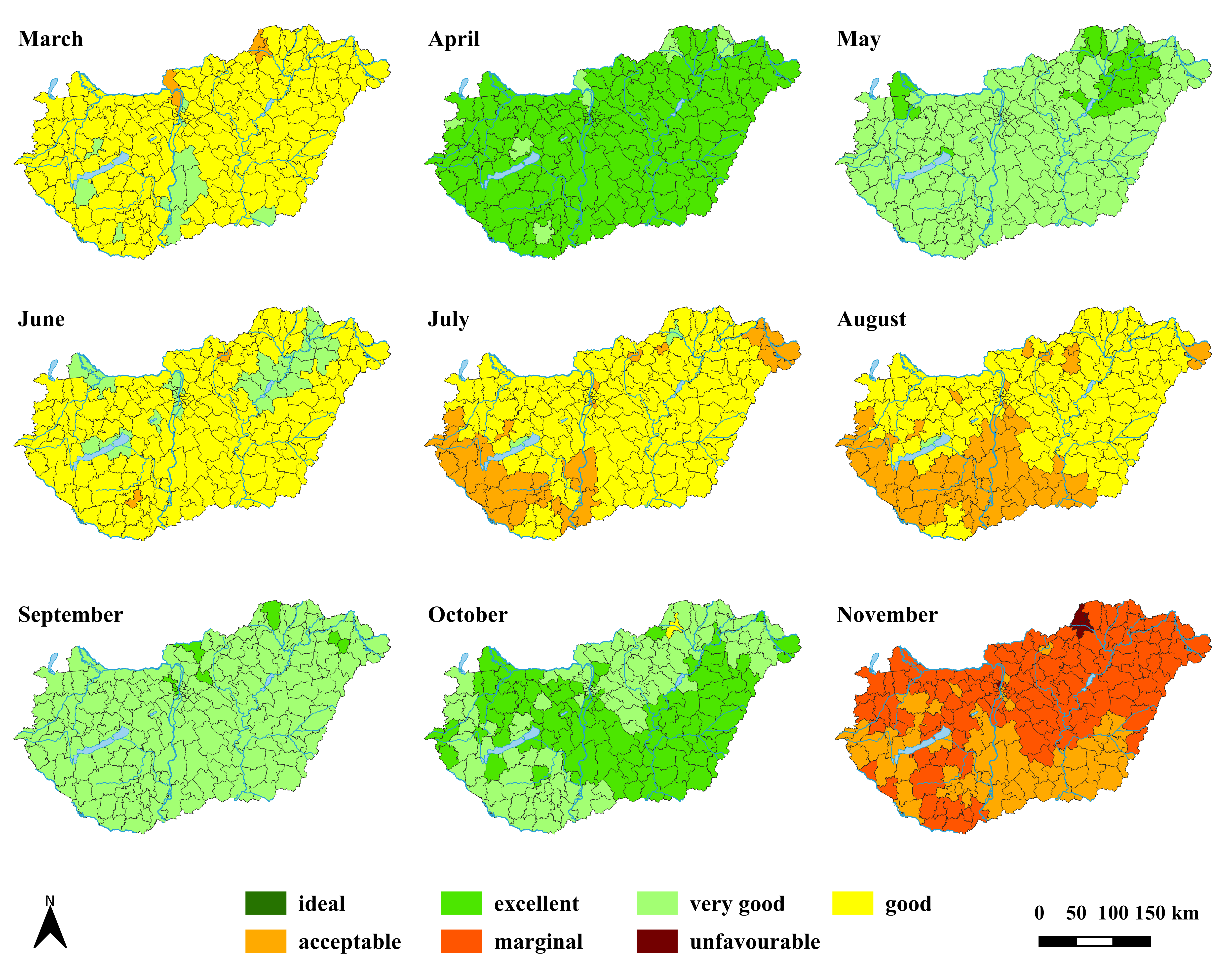


**Fig. S17** Spatial distribution of monthly mTCI ratings by district for the period 2041–2070 based on the maximum values of simulated results
